# Cellular immunity to SARS-CoV-2 following intrafamilial exposure in seronegative family members

**DOI:** 10.3389/fimmu.2023.1248658

**Published:** 2023-08-29

**Authors:** Cecilia Jay, Emily Adland, Anna Csala, Christina Dold, Matthew Edmans, Carl-Philipp Hackstein, Anni Jamsen, Nicholas Lim, Stephanie Longet, Ane Ogbe, Oliver Sampson, Donal Skelly, Owen B. Spiller, Lizzie Stafford, Craig P. Thompson, Lance Turtle, Ellie Barnes, Susanna Dunachie, Miles Carroll, Paul Klenerman, Chris Conlon, Philip Goulder, Lucy C. Jones

**Affiliations:** ^1^ Nuffield Department of Medicine, University of Oxford, Oxford, United Kingdom; ^2^ Department of Paediatrics, University of Oxford, Oxford, United Kingdom; ^3^ Oxford Vaccine Group, University of Oxford, Oxford, United Kingdom; ^4^ Wellcome Centre for Human Genetics, University of Oxford, Oxford, United Kingdom; ^5^ Peter Medawar Building for Pathogen Research, University of Oxford, Oxford, United Kingdom; ^6^ Nuffield Department of Clinical Neurosciences, University of Oxford, Oxford, United Kingdom; ^7^ Oxford University Hospitals, University of Oxford, Oxford, United Kingdom; ^8^ Division of Infection and Immunity, Cardiff University School of Medicine, Cardiff, United Kingdom; ^9^ Institute of Infection, Veterinary and Ecological Sciences, University of Liverpool, Liverpool, United Kingdom; ^10^ National Institute for Health and Care Research (NIHR) Oxford Biomedical Research Centre, Oxford University Hospitals National Health Service (NHS) Foundation Trust, Oxford, United Kingdom

**Keywords:** SARS-CoV-2, COVID-19, exposed seronegative, family, T-cells

## Abstract

**Introduction:**

Family studies of antiviral immunity provide an opportunity to assess virus-specific immunity in infected and highly exposed individuals, as well as to examine the dynamics of viral infection within families. Transmission of SARS-CoV-2 between family members represented a major route for viral spread during the early stages of the pandemic, due to the nature of SARS-CoV-2 transmission through close contacts.

**Methods:**

Here, humoral and cellular immunity is explored in 264 SARS-CoV-2 infected, exposed or unexposed individuals from 81 families in the United Kingdom sampled in the winter of 2020 before widespread vaccination and infection.

**Results:**

We describe robust cellular and humoral immunity into COVID-19 convalescence, albeit with marked heterogeneity between families and between individuals. T-cell response magnitude is associated with male sex and older age by multiple linear regression. SARS-CoV-2-specific T-cell responses in seronegative individuals are widespread, particularly in adults and in individuals exposed to SARS-CoV-2 through an infected family member. The magnitude of this response is associated with the number of seropositive family members, with a greater number of seropositive individuals within a family leading to stronger T-cell immunity in seronegative individuals.

**Discussion:**

These results support a model whereby exposure to SARS-CoV-2 promotes T-cell immunity in the absence of an antibody response. The source of these seronegative T-cell responses to SARS-CoV-2 has been suggested as cross-reactive immunity to endemic coronaviruses that is expanded upon SARS-CoV-2 exposure. However, in this study, no association between HCoV-specific immunity and seronegative T-cell immunity to SARS-CoV-2 is identified, suggesting that de novo T-cell immunity may be generated in seronegative SARS-CoV-2 exposed individuals.

## Introduction

The emergence of severe acute respiratory syndrome coronavirus 2 (SARS-CoV-2) offered an unprecedented opportunity to study immunity to a novel pathogen in an immunologically naïve population. Early in the coronavirus disease 19 (COVID-19) pandemic, ongoing lockdowns provided an opportunity to examine SARS-CoV-2-specific immune responses in family households where transmission between close contacts was common and social mixing outside of households was limited. In 2020, a significant degree of our understanding of SARS-CoV-2 transmission risk arose from small case studies, many of which involved intrafamilial transmission (1–3). As well as querying transmission dynamics, another benefit of family studies of SARS-CoV-2 infection is the opportunity to interrogate age- and sex-related determinants of immunity.

The role of age in disease severity and immune response is well characterized: low-grade inflammation, thymic aging, and reduced cellular functionality in older individuals associated with “immunosenescence” promote worse disease outcomes for many infections in the elderly (4). Children experience low rates of COVID-19 mortality, perhaps owing to their increased numbers of naïve T cells, high degree of exposure to related respiratory viruses, and reduced inflammatory phenotypes (5). Furthermore, sex-specific differences in immune response owing to the location of immune genes on sex chromosomes, the immunomodulatory effects of sex hormones, and differential cytokine profiles in male and female patients generally promote greater adaptive immune responses and stronger autoimmune phenotypes in female patients (6). These established trends raised the possibility that weaker adaptive immune responses may contribute to the worse disease outcomes and higher mortality identified in male patients with COVID-19 (7). Studying SARS-CoV-2-specific immunity in families provided an opportunity to test these hypotheses in male and female patients of diverse ages with differing levels of exposure to SARS-CoV-2.

Additionally, it has been demonstrated that individuals highly exposed to SARS-CoV-2, as well as other viruses such as hepatitis C virus (HCV), can generate pathogen-specific cellular immunity in the absence of specific antibodies (8–10) and, in the case of SARS-CoV-2, that these cellular responses are of higher magnitude than would be expected from cross-reactivity (such as with the endemic human coronaviruses [HCoVs] OC43, HKU1, 229E, and NL63 that circulate widely in the UK, causing common cold symptoms) in unexposed individuals. The source of elevated cellular responses in SARS-CoV-2-exposed individuals is believed to be expansion of pre-existing memory responses to HCoVs that abort SARS-CoV-2 infection before seroconversion can occur (9). However, this seronegative cellular immunity has not been extensively explored. Questions that remain include the following: How much exposure is required to generate this immunity? Are these responses wholly cross-reactive memory responses against HCoVs or does seronegative exposure also generate *de novo* responses to non-conserved T-cell epitopes? Are T-cell responses equally distributed among seronegative adults and children?

Here, a large dataset of SARS-CoV-2-infected and -exposed families was generated through recruitment of families in Oxford, London, and Cardiff during the winter of 2020 and the spring of 2021. As well as assessing transmission dynamics of SARS-CoV-2, the aim of this study was to characterize immunity to primary SARS-CoV-2 infection in family members, with a focus on differences in age and sex. Furthermore, we examine the quality and likely source of specific T-cell immunity in seronegative individuals.

## Materials and methods

### Ethics

Venous blood samples were donated from October 2020 to March 2021. Eligible participants were individuals aged 6 years or above who either had experienced symptoms of COVID-19 or had a family member who had. Samples from Wales were collected as part of the CROWN study, where index patients from the CROWN study were recruited along with their families. Families from Oxford and London were recruited by word of mouth from different neighborhoods, whereas families in Cardiff were recruited through GP visits. Families were visited and blood was collected only once from each family. It was generally possible to collect samples from all members of a household, except on some occasions from children whose parents did not consent or from whom sufficient blood samples were difficult to obtain for practical reasons. As this study was carried out before widespread PCR and lateral flow testing and vaccines became available, history of SARS-CoV-2 infection was determined by anti-Spike (S) immunoglobulin G (IgG) enzyme-linked immunosorbent assay (ELISA) above a threshold of 10 ELISA units (EU). ELISAs were carried out by collaborators at the Oxford Vaccine Group on 232/264 individuals. Written informed consent was obtained from patients; ethical approval was granted by the Central University Research Ethics Committee (CUREC R71346/RE001) and Brighton and Sussex HRA Research Ethics Committee (IRAS reference 269506).

### Sample collection and processing

Whole blood EDTA samples were transported from site of sampling to the laboratory and processed within 6 h. Isolation of peripheral blood mononuclear cells (PBMCs) and plasma was carried out as described elsewhere (11). Briefly, PBMCs were separated by density gradient centrifugation using Lymphoprep (1.077 g/ml, Stem Cell Technologies). PBMCs were washed twice in RPMI 1640 (Sigma, USA) with 10% heat-inactivated fetal calf serum, 1% penicillin/streptomycin (Sigma, USA), and 2 mM L-glutamine (Sigma, USA). Plasma was spun at 2,000*g* for 10 min to remove platelets. Cells were resuspended in RPMI and counted using a Muse Cell Analyser (Luminex, USA). Assays were run on fresh PBMCs, or samples were cryopreserved as 0.5-ml aliquots in 90% fetal calf serum with 10% dimethylsulfoxide (DMSO) and stored at −80°C for later use.

### Serological assays

As well as ELISA, total IgG targeting SARS-CoV-2 S, receptor-binding domain (RBD), and nucleocapsid (N) was quantified using a Meso Scale Diagnostics (MSD) v-plex immunoassay “Coronavirus panel 3” (MSD, USA) according to the manufacturer’s protocol and as described elsewhere (12). Plates were incubated in Blocker A for 30 min at room temperature with 700 rpm shaking. Serum was diluted 1/1,000 and 1/10,000 in Diluent 100. A seven-point standard curve of MSD reference standard starting at 1/10 was prepared in duplicate; three internal controls and one in-house control of COVID-19 convalescent serum was used. Diluent 100 was used as a negative control. Plates were washed three times in MSD Wash buffer and samples and controls were added to the plate. Plates were incubated for 2 h at room temperature with shaking. Plates were washed three times again before addition of detection antibody and incubation for 1 h with shaking. Plates were washed three times, MSD Gold read buffer was added, and plates were immediately read with a MESO QuickPlex SQ 120 (MSD, USA). Data were analyzed in MSD Discovery Workbench. The threshold for S, RBD, and N positivity (S: 1,160 AU/ml, RBD: 1,169 AU/ml, N: 3,874 AU/ml) was taken from analyses of pre-pandemic sera (12).

Total IgG targeting SARS-CoV-2 S2 was quantified using indirect ELISA. S2 antigen (Sino Biological, China) was diluted to 1 μg/ml in PBS and used to coat 535 Nunc-Immuno 96-well plates (Thermo Fisher Scientific, USA) at 4°C overnight. Plates were washed three times in PBS with 0.1% Tween before blocking with Casein Buffer for 1 h at room temperature. Serum was diluted in Casein Buffer 1/500 and plated in duplicate alongside a 10-point standard curve of pooled COVID-19 convalescent sera beginning at 1/25. Casein Buffer was used as a blank. Plates were incubated at room temperature for 2 h and washed six times in PBS with 0.1% Tween. Goat anti-human IgG conjugated to alkaline phosphatase (Sigma, USA) was diluted to 1/1,000 in Casein Buffer and added to plates for 1 h at room temperature. Plates were washed six times in PBS with 0.1% Tween. 4-Nitrophenyl phosphate in diethanolamine buffer (Pierce, UK) was added and plates were incubated for 15 min. Plates were read on an ELx800 microplate reader at 405-nm absorbance (Cole Parmer, UK). Concentrations were calculated by mapping a line of best fit onto the standard curve, then substituting mean absorbance values for each sample into the line equation. This was then multiplied by the dilution factor of 500 to give the final result.

### Proliferation assay

T-cell proliferation was quantified using a CellTrace Violet proliferation assay as described elsewhere (11). Frozen PBMCs were thawed in RPMI with 10% fetal calf serum, 1% penicillin/streptomycin, and 2 mM L-glutamine. After washing twice with PBS, cells were stained with CellTrace Violet at 2.5 μM (Life Technologies, USA) for 10 min at room temperature. Cold fetal calf serum was added to quench the stain. Cells were plated in 96-well round-bottom plates at 250,000 cells per well. Pools of 18-mer peptides overlapping by 10 amino acids spanning the whole SARS-CoV-2 genome [S1, S2, membrane (M), N, open reading frame (ORF) 3, ORF8, non-structural protein (NSP) 1 + 2, NSP3A, NSP3B, NSP3C, NSP4, NSP5 + 6, NSP7-11, NSP12A, NSP12B, NSP13, NSP14, and NSP15 + 16]) were added to wells at a final concentration of 1 μg/ml. RPMI was used as a negative control, and phytohemagglutinin L (Sigma, USA) was used as a positive control at a final concentration of 2 μg/ml. Plates were incubated for 7 days at 37°C, 5% CO_2_, 95% humidity with a hemimedia change on day 4. Cells were then washed in PBS and stained for CD4, CD8, and CD3 in PBS with fluorochrome-conjugated antibodies (BioLegend, USA). LIVE/DEAD Aqua was used to stain dead cells (Thermo Fisher Scientific, USA). Cells were fixed in 4% PFA (Sigma, USA) at 4°C for 10 min, washed in PBS, and stored at 4°C in the dark. Samples were acquired the next day on a MACSQuant X (Miltenyi, Germany) and analyzed in FlowJo. Cutoff for positive responses was set at 1% proliferation as determined previously (11).

### Neutralization assay

Neutralizing antibody (nAb) titers were calculated using a SARS-CoV-2 lentivirus-based pseudovirus assay displaying a codon-optimized SARS-CoV-2 S protein (National Center for Biotechnology Information [NCBI] reference sequence: YP_009724390.1) as described elsewhere (13). Briefly, HEK293 T/17 cells were cultured in complete medium [Dulbecco’s Modified Eagle Medium (DMEM) supplemented with 10% fetal calf serum, 1% penicillin/streptomycin, and 1% L-glutamine] and incubated at 37°C/5% CO_2_. Pseudotyped viruses were produced by transfecting HEK293T cells with 1 μg of codon-optimized S, 1 μg of gag/pol, and 1.5 μg of a luciferase reporter in a plasmid-OptiMem solution.

For the microneutralization assay, a transfection mix was prepared using 2,500 ng of ACE2, 250 ng of TMPRSS2, 1 ml of OptiMem, and 9 μl of Fugene (ProMega, USA) as part of a plasmid-media mix. Cells (10 ml) were transfected with the plasmid-media mix 24 h before the assay. Five microliters of serum and 95 μl of complete media were added to columns 1 and 7 in a 96-well white opaque culture plate. Complete media (50 μl) was added to columns 2 to 6 and 8 to 12 and serum was serially diluted across the plate in a 1:2 dilution. Pseudotyped virus (50 μl) was added to wells, mixed, and incubated for 2 h at 37°C. Each plate contained six positive controls wells (no serum) and six negative control wells (no pseudovirus). A total of 10^4^ plasmid transfected HEK293 T/17 cells were added to each well and incubated for 48 h at 37°C/5% CO_2_. Supernatant was removed using vacuum filtration. Bright Glo (Promega, USA) was diluted 1:1 with sterile PBS. Bright Glo-PBS mixture (50 μl) was added to each well and allowed to lyse for 5 min, after which luciferase activity was measured using a GloMax Luminometer (ProMega, US). Data were analyzed using Microsoft Excel and GraphPad Prism.

### Statistical analysis

All statistical comparisons, logistic regression, and multivariable linear regression were performed in GraphPad Prism 9.0. For pairwise comparisons, two-tailed Mann–Whitney tests were used for unpaired data. For multiple comparisons, Kruskal–Wallis tests with Dunn’s multiple comparisons test were used. For correlations, Spearman’s rank tests were used.

## Results

### Cohort

**Figure 1:**
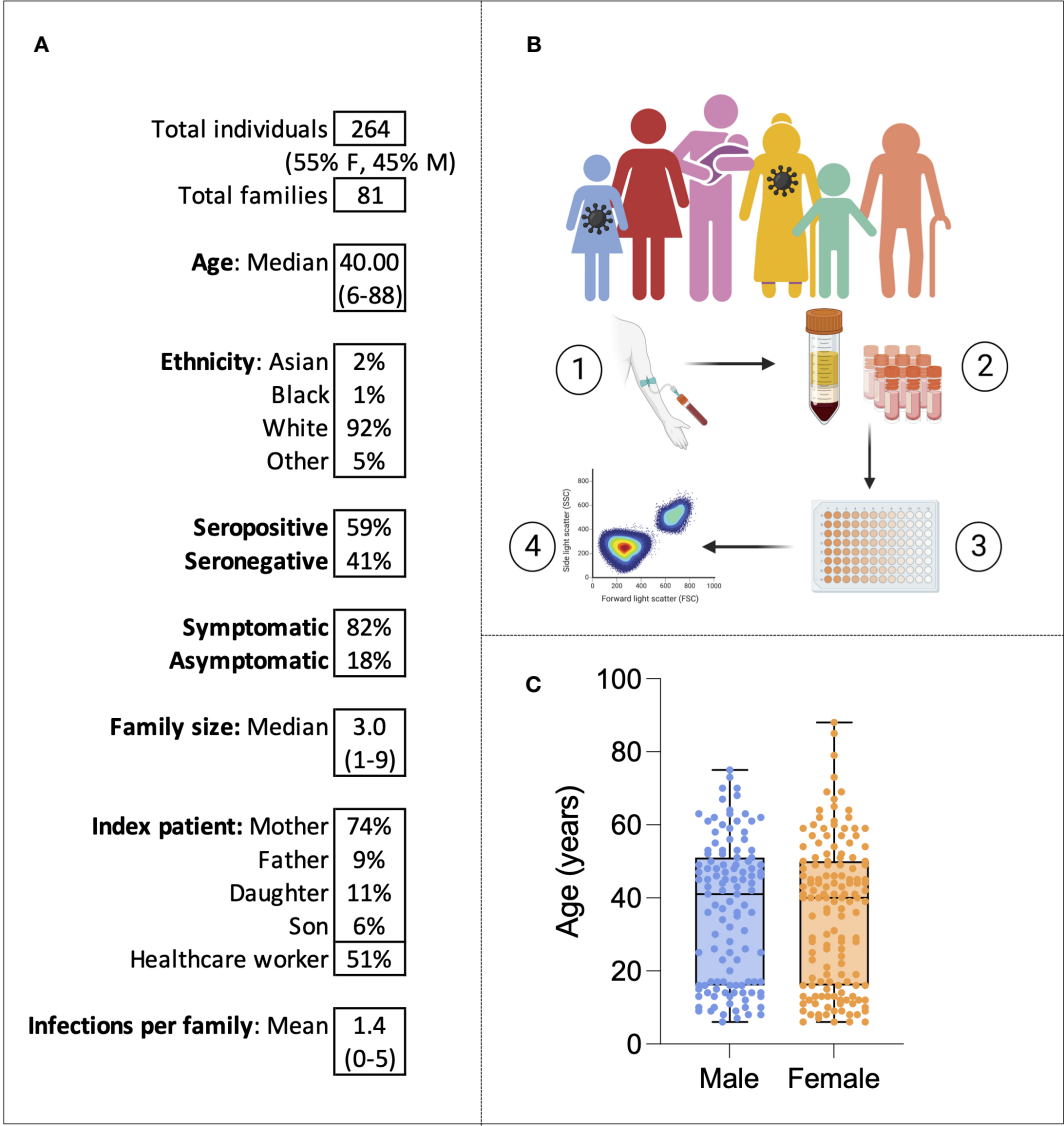
The family cohort. Characteristics of the cohort **(A)**. Graphical representation of the study: sampling of infected individuals and their family members, PBMC and serum isolation, assays, and analysis **(B)**. Age distribution of the cohort by sex **(C)**. Virus symbols indicate exemplar seropositive individuals.

Families were eligible for the study if at least one family member had experienced symptoms of COVID-19 between October 2020 and March 2021. Over a period of 6 months of recruitment, a total of 264 individuals belonging to 81 families were recruited from Oxford, London, and Cardiff (**Figure 1A**). From each study participant, whole-blood EDTA samples were taken, and PBMCs and plasma were isolated and cryopreserved for later use (**Figure 1B**). Fifty-five percent of individuals were women, and the median age was 40, ranging from 6 to 88 years of age (**Figure 1C**). The cohort was predominantly white British (92%). Fifty-two percent (*n* = 136) of individuals were seropositive for SARS-CoV-2 anti-S IgG at the time of enrolment and 94% (*n* = 248) were unvaccinated; vaccinated individuals were excluded from later analysis. A total of 19 individuals self-reported pre-existing medical conditions, including Graves’ disease, asthma, gout, epilepsy, type 2 diabetes, Crohn’s disease, atrial fibrillation, depression, hypertension, obesity, and celiac disease. In most families (74%), the index patient recruited to the study was the mother, and in 51% of families, the index patient was a healthcare worker (HCW). Of the 136 individuals seropositive for SARS-CoV-2 at enrolment, 127 self-reported symptoms. All symptomatic individuals experienced mild disease and none were hospitalized. The following symptoms were self-reported: cough, fever, anosmia, gastrointestinal symptoms, sore throat, fatigue, myalgia, and a runny nose. Among the 127 individuals that self-reported symptoms, sampling occurred a mean of 228 days (7.6 months) after symptom onset.

### Transmission dynamics

To examine the dynamics of SARS-CoV-2 infection within and between families, families with similar patterns of seropositivity were identified and grouped into seven family types (**Supplementary Figure 1**). These included the following: all seropositive (18 families), all seronegative (6 families), seropositive father/adult male (3 families), seropositive mother/adult female (17 families), seropositive children only (5 families), serodiscordant parents with at least one seropositive child (6 families), and double-seropositive parents with at least one seropositive child (7 families). Interestingly, the rarest groups, therefore, were seropositive children only and adult seropositive male only. All double-seropositive parents had at least one seropositive child. Overall, the families represented a diverse group, indicating that intrafamilial transmission can occur through multiple routes.

To assess whether the number and proportion of individuals infected within a family increased someone’s risk of infection, the following logistic regression was carried out:


Infected?~Intercept+Proportion infected+Family size

The model demonstrated that, holding family size constant, the odds of an individual becoming infected increased by 15% (95% CI [1.04 to 1.28]) for every 10% increase in the percentage of family members infected (*p* = 0.001). There was no increased infection risk associated with family size (*p* = 0.12).

### Immune dynamics within families

**Figure 2:**
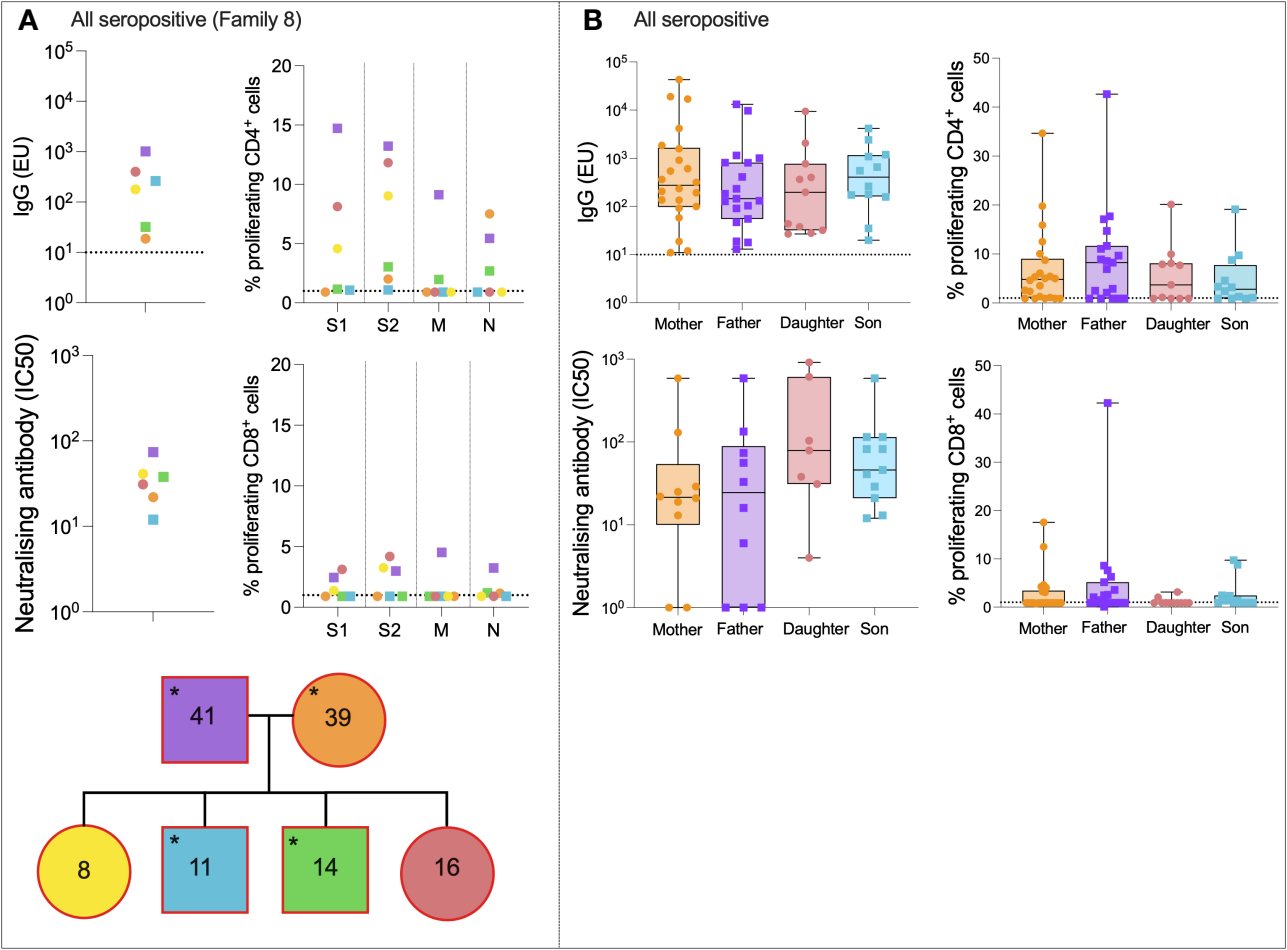
All seropositive families. Anti-S IgG, nAb responses, CD4+ T-cell responses, and CD8+ responses in a seropositive family group consisting of mother, father, two sons, and two daughters **(A)**. IgG, nAb, and T-cell responses in all individuals from the “All seropositive” family type **(B)**. Male patients are squares; female patients are circles. Seropositive individuals are outlined in red. Asterisks refer to symptomatic individuals. Proliferation values below 1% were given nominal values of 0.9%. Dotted lines refer to cutoffs as determined previously (11, 12).

To compare the immune dynamics of SARS-CoV-2 infection between family members, first, a representative case study was examined in detail. A fully seropositive family (Family 008), consisting of a 39-year-old mother, a 41-year-old father, two daughters, and two sons, is shown in **Figure 2A**. All individuals made IgG responses to SARS-CoV-2 S; the response of greatest magnitude was the father (1,011 EU), and the response of lowest magnitude was the mother (18 EU). The greatest nAb response (IC_50_ = 74) and S1-specific CD4+ T-cell response (14% proliferation) also belonged to the father. All individuals made CD4+ T-cell responses to either S1 or S2 pools; CD8+ responses were weaker.

To compare immune responses between all mothers, fathers, daughters, and sons from the “all seropositive” group, and to assess any age- or sex-related differences in immunity, IgG, nAb titer, and S1-specific CD4+ and CD8+ T-cell responses were compared (**Figure 2B**). Notably, there was no difference in IgG, nAb response, or S1-specific CD4+ or CD8+ T-cell response between any of the family members in this group (IgG: *p* = 0.65, nAb: *p* = 0.22; T cell: *p* = 0.76, Kruskal–Wallis test with Dunn’s multiple comparisons).

**Figure 3:**
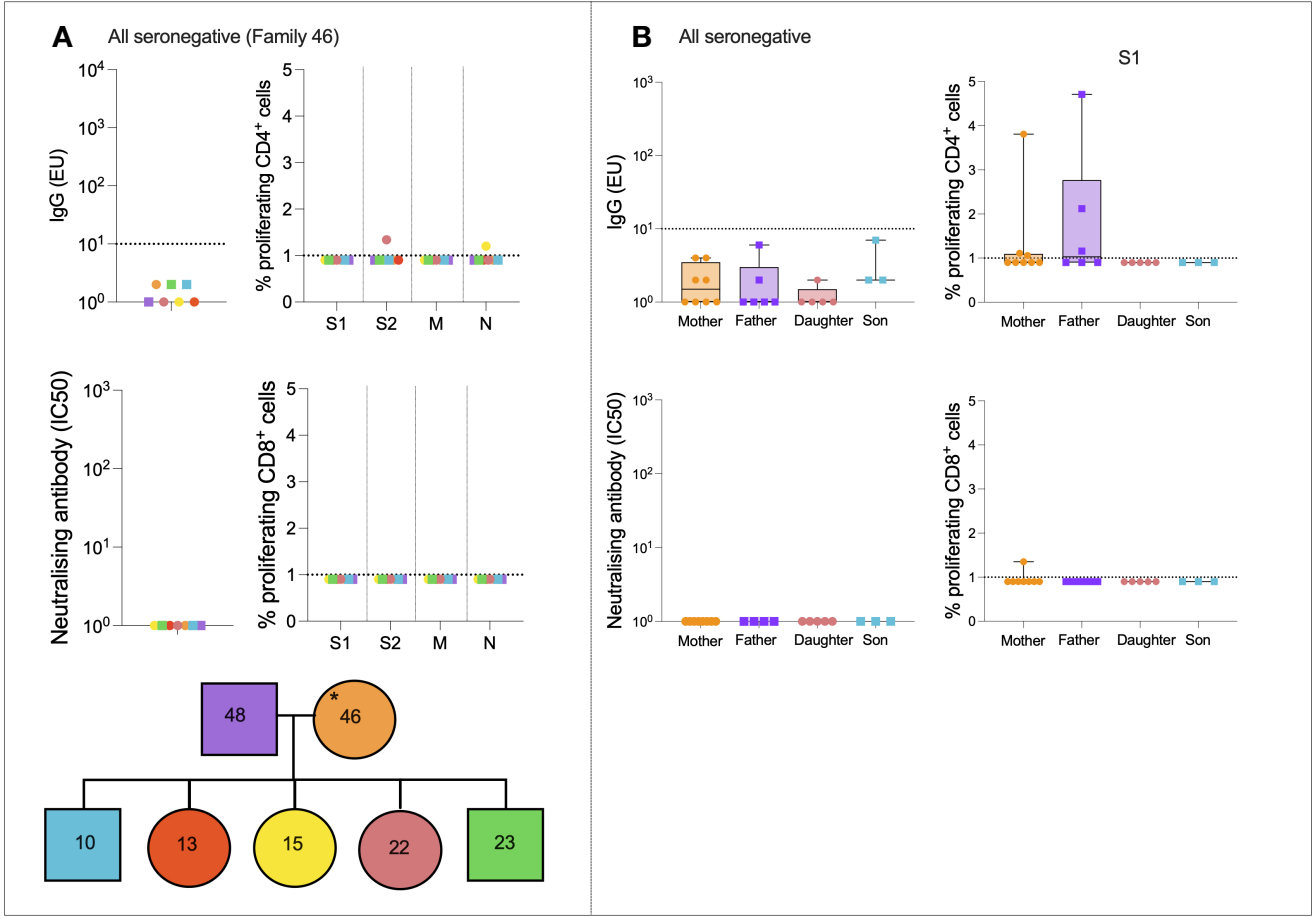
All seronegative families. Anti-S IgG, nAb responses, CD4+ T-cell responses, and CD8+ responses in a seronegative family group consisting of mother, father, two sons, and three daughters **(A)**. IgG, nAb, and T-cell responses in all individuals from the “All seronegative” family type **(B)**. Male patients are squares; female patients are circles. Asterisks refer to symptomatic individuals. Proliferation values below 1% were given nominal values of 0.9%. Dotted lines refer to cutoffs as determined previously (11, 12).

We next assessed SARS-CoV-2-specific immune dynamics in “all seronegative” families to identify potential cross-reactive immunity from endemic HCoVs. Although these individuals were seronegative, in order to be recruited onto the study, at least one individual self-reported symptoms of COVID-19. A representative case study family was first assessed (**Figure 3A**). Family 046 consisted of a mother, father, three daughters, and two sons who generated no IgG or nAbs to SARS-CoV-2, as defined (**Figure 3A**). T-cell responses were absent in most family members, although the 22-year-old daughter generated a weak CD4+ response to S2 (1.3% proliferation), and the 15-year-old daughter generated a weak CD4+ response to N (1.2% proliferation).

To determine if any form of SARS-CoV-2-specific immunity was present in members of the “all seronegative” group, and to uncover any age- and sex-specific trends, IgG, nAbs, and T-cell responses were compared between all mothers, fathers, daughters, and sons in the “all seronegative” group (**Figure 3B**). Of note, some mothers and fathers generated CD4+ T-cell responses to S1. However, no children generated a CD4+ or CD8+ response to S1, raising the question of whether pre-existing cellular immunity may be found at higher levels in older individuals.

Case study families of mixed-serostatus families were also assessed (**Supplementary Figures 2**, **3**). Cross-reactive cellular immunity appeared widespread in seronegative individuals who had been exposed to SARS-CoV-2 through infected family members. These T-cell responses were present in both seronegative adults and seronegative children.

The cohort consisted of individuals who experienced only mild symptoms; however, these symptoms were self-reported at the time of sampling and therefore individuals could be classed as symptomatic or asymptomatic. Symptomatic individuals had significantly greater magnitude S1-, M-, and N-specific CD4+ T-cell responses (*p* = 0.002, *p* = 0.004, and *p* = 0.009, respectively; Mann–Whitney tests) but anti-S IgG and nAb responses did not differ significantly between symptomatic and asymptomatic individuals.

### Age- and sex-specific trends in immunity

To analyze more closely if age and sex had an impact on the magnitude of humoral or cellular immune responses, two multivariable linear regressions were run. Model 1 calculated the effects of age, sex, and days since symptom onset on total IgG response in seropositive individuals. The fitted regression model was:


IgG=2,744−15.78*(Age)−195.2*(Sex[Male])–5.808*(Days since symptoms)

The overall regression was statistically significant (*R* ^2^ = 0.22, *F*(3, 35) = 3.29, *p* = 0.03). It was found that neither age (β = −15.78, *p* = 0.26) nor male sex (β = −195.2, *p* = 0.67) significantly predicted IgG response. However, days since symptoms (β = −5.808, *p* = 0.017) was negatively associated with IgG response, indicating some waning of humoral immunity over time.

Model 2 calculated the effects of age, sex, and days since symptom onset on S1-specific CD4+ T-cell responses in seropositive individuals. The fitted regression model was:


$$\begin{array}{c} \text{CD}4+\text{response}=4.82+0.23*\left(\text{Age}\right)+8.57*\\ \left(\text{Sex}\left[\text{Male}\right]\right)–0.037*\left(\text{Days since symptoms}\right) \end{array}$$

The overall regression was statistically significant (*R* ^2^ = 0.36, *F*(3, 35) = 6.69, *p* = 0.001). It was found that age (β = 0.23, *p* = 0.008), male sex (β = 8.57, *p* = 0.004), and days since symptoms (β = −0.037, *p* = 0.01) were significantly associated with CD4+ response. This indicated that older men generated stronger CD4+ T-cell responses to S1.

Finally, the correlation between anti-S IgG and CD4+ T-cell response was calculated for male and female patients separately. In female patients only, IgG was weakly correlated with S1-specific T cells (*r* = 0.3, *p* = 0.006) and S2-specific T cells (*r* = 0.26, *p* = 0.02).

### Cross-reactive cellular immunity in exposed seronegative individuals

It was hypothesized that that exposure to SARS-CoV-2 through infected family members might expand T-cell immunity to SARS-CoV-2 in seronegative individuals, as has been demonstrated elsewhere (9, 10). To test this hypothesis, individuals were split into three groups—seropositive (*n* = 121), exposed seronegative (ESN, *n* = 72) who had at least one seropositive family member, and unexposed seronegative (USN, *n* = 24) with no history of infection, no seropositive family members, and at least one symptomatic family member.

**Figure 4:**
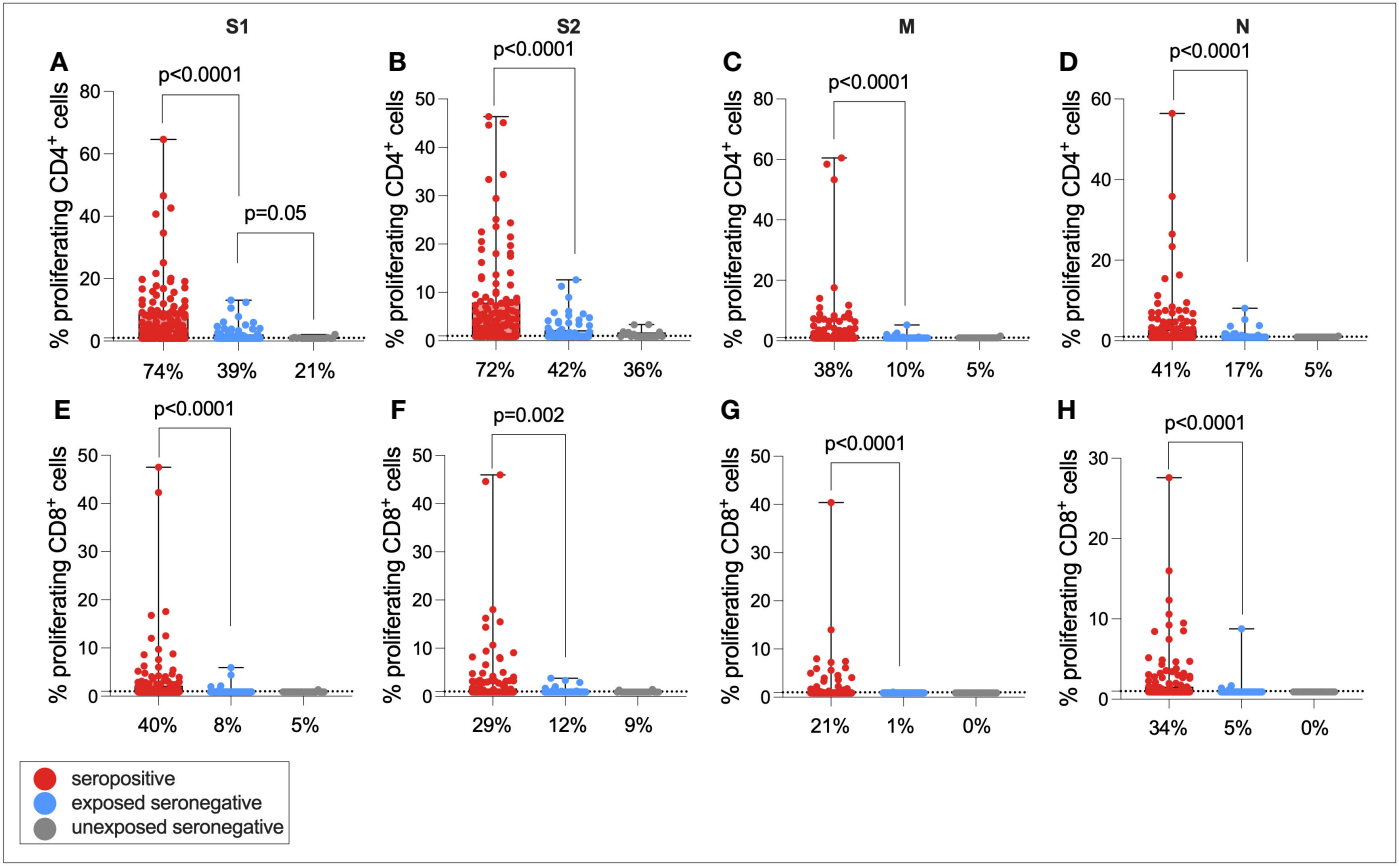
Cellular immunity in seropositive, ESN, and USN individuals. CD4+ T-cell responses to SARS-CoV-2 S1 **(A)**, S2 **(B)**, M **(C)**, and N **(D)** as measured by proliferation assay in seropositive individuals (red), ESNs (blue), and USNs (gray). CD8+ T-cell responses to S1 **(E)**, S2 **(F)**, M **(G)**, and N **(H)** as measured by proliferation assay. *p*-values refer to Mann–Whitney test values. Data points below 1% were given nominal values of 0.9%. Dotted lines refer to cutoffs as determined previously (11). Percentages refer to the number of individuals with responses above 1% proliferation.

T-cell responses were then compared between seropositive individuals, ESNs, and USNs (**Figure 4**). As expected, seropositive individuals generated significantly higher magnitude CD4+ responses compared to ESNs (all *p* < 0.0001, Mann–Whitney tests), and significantly greater CD8+ responses (all *p* < 0.0001, Mann–Whitney tests). Of note, ESNs generated stronger S1-specific CD4+ T-cell responses than USNs (*p* = 0.05, Mann–Whitney test) (**Figure 4A**), and this trend held for S2, M, and N, although not significantly (S2: *p* = 0.6, M: *p* = 0.5, N: *p* = 0.16, Mann–Whitney tests). There was also a trend of greater CD8+ responses to S1 in ESNs compared to USNs, although again this was not significant (*p* = 0.49, Mann–Whitney test) (**Figure 4E**).

One possibility is that the same ESNs who generated CD4+ responses to S1 also generated responses to S2, as well as also generating low-level nAb and IgG responses. To test these hypotheses, the correlation between S1-specific CD4+ responses and S2-specific response, nAb response, and IgG response was calculated in ESNs only (**Supplementary Figure 4**). CD4+ responses to S1 and S2 were correlated (*r* = 0.39, *p* = 0.0008), but responses to S1 were not correlated with nAb or IgG responses, suggesting that ESNs with high magnitude S1-specific T-cell immunity had not simply generated antibody responses that had waned to levels below the limit of detection but remained above zero. Classifying ESN responses in a binary way, irrespective of magnitude, there was a significant relationship between being an S1 responder and being an S2 responder (proliferation>1%) (*p* = 0.007, Fisher’s exact test) but not between being an S1 responder and a low-level IgG (EU > 1) or nAb (IC_50_ > 0) responder.

**Figure 5:**
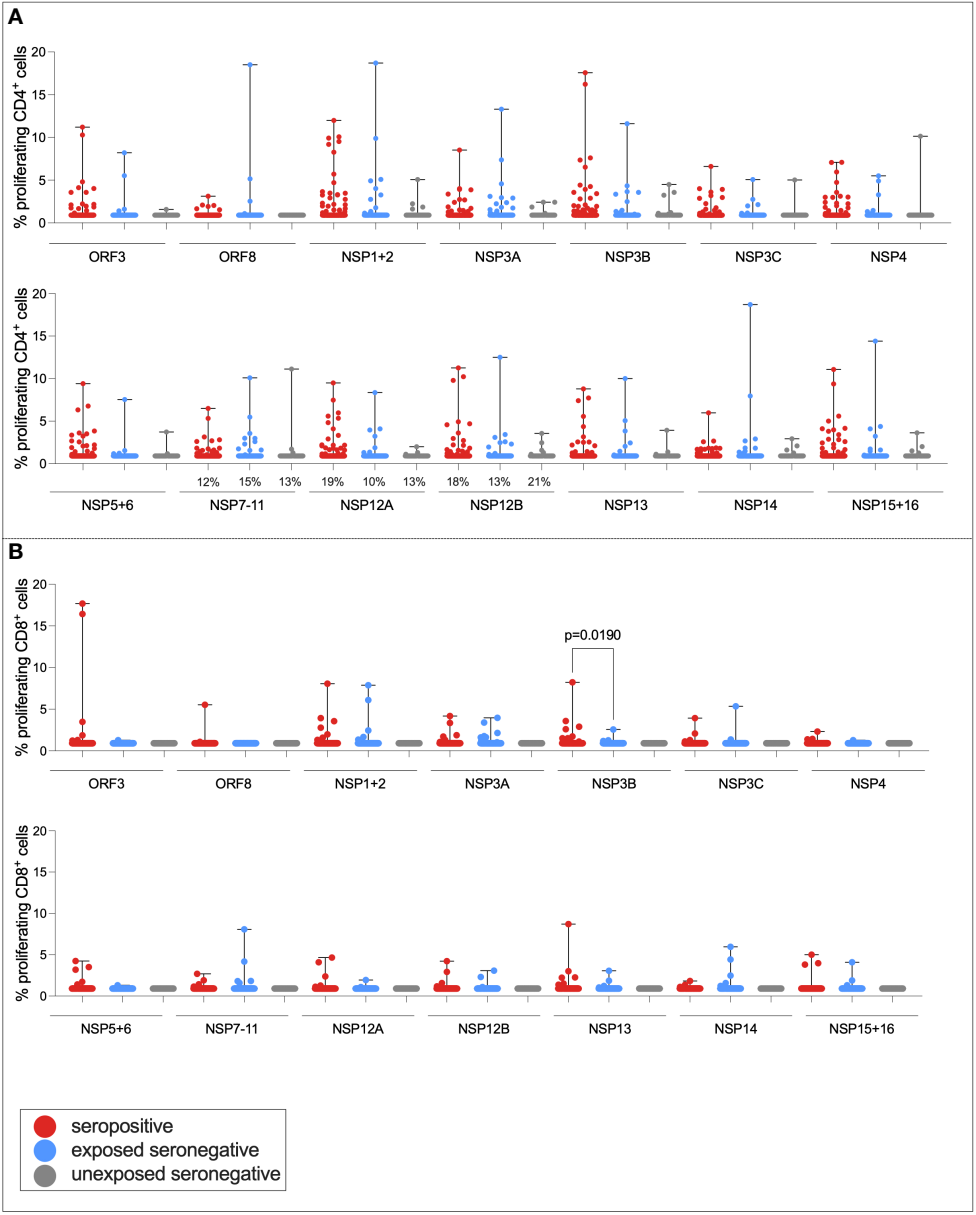
T-cell responses to NSPs in ESN individuals. CD4+ T-cell responses targeting SARS-CoV-2 NSPs in seropositive (red), ESN (blue), and USN (gray) individuals **(A)**. CD8+ T-cell responses targeting SARS-CoV-2 NSPs in seropositive, ESN and USN individuals **(B)**. Percentages refer to the number of individuals with responses above 1% proliferation, specifically for the RTC region of the genome. *p*-values refer to Kruskal–Wallis test values with Dunn’s multiple comparisons. Data points below 1% were given nominal values of 0.9%.

It has been demonstrated that a target of T-cell responses in ESNs is the replication–transcription complex (RTC), an early-translated protein that is highly conserved between SARS-CoV-2 and endemic HCoVs (consisting of non-structural protein [NSP] 7, NSP12, and NSP13) (9). To test the hypothesis that ESN individuals generate enhanced responses to the RTC compared to USNs, both CD4+ and CD8+ T-cell responses to pools of overlapping peptides spanning the rest of the SARS-CoV-2 genome (ORF3, ORF8, NSP1 + 2, NSP3A, NSP3B, NSP3C, NSP4, NSP5 + 6, NSP7-11, NSP12A, NSP12B, NSP13, NSP14, and NSP15 + 16) were compared between seropositive, ESN, and USN individuals (**Figure 5**). Responses were generally stronger in seropositive individuals than ESNs, reaching significance in CD8+ responses to NSP3B (*p* = 0.02, Kruskal–Wallis test with Dunn’s multiple comparisons). However, there was no significant difference in responses to the RTC region between ESN and USN individuals, and no obvious trend of greater responses in ESNs. To test for the presence of a T-cell response rather than magnitude, Fisher’s exact test identified no association between presence of a T-cell response to NSP7-11, NSP12A, or NSP12B and belonging to ESN vs. USN groups (*p* > 0.99, *p* = 0.7, and *p* = 0.3, respectively).

**Figure 6:**
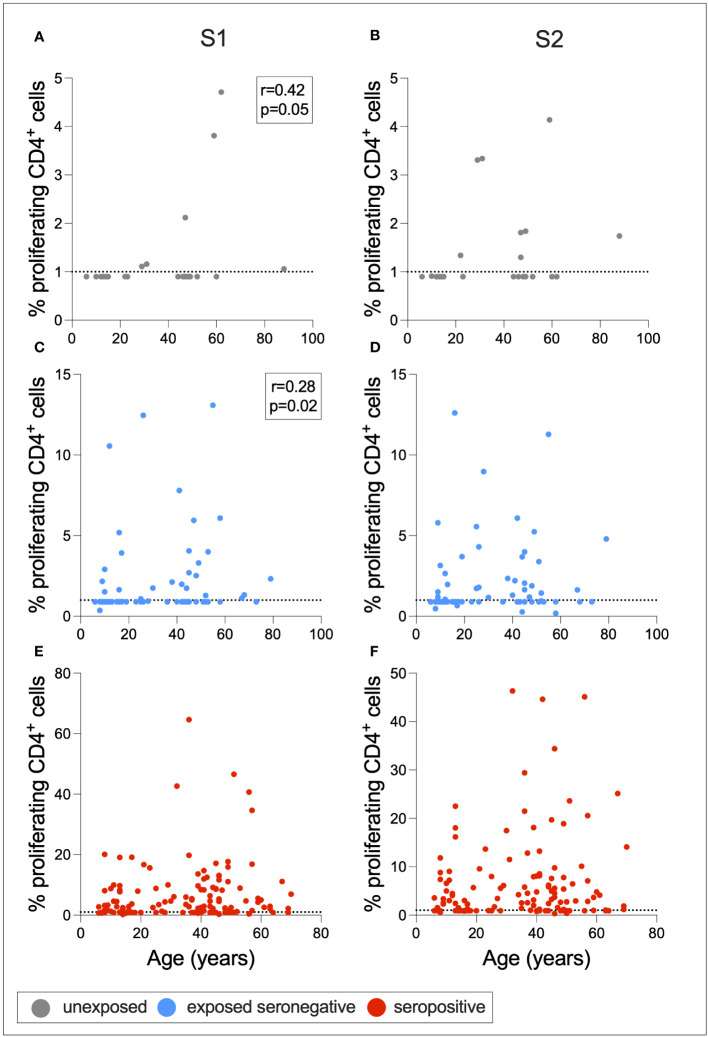
Associations between age and T-cell response. Correlations between age and S1- or S2-specific CD4+ T-cell response in USNs (**A, B** gray), ESNs (**C, D** blue), and seropositive individuals (**E, F** red). *R-* and *p*-values refer to Spearman rank test values. Data points below 1% were given nominal values of 0.9%. Dotted lines refer to cutoffs as determined previously (11).

To determine whether cross-reactive immunity is found at higher levels in seronegative adults compared to children, and whether exposure to SARS-CoV-2 induces T-cell immunity in otherwise T-cell negative children, Spearman rank correlations were calculated between age in years and S1- or S2-specific CD4+ T-cell response for ESNs, USNs, and seropositive individuals (**Figure 6**). There was a positive correlation between age and S1-specific T cells in USNs (*r* = 0.42, *p* = 0.05) (**Figure 6A**) and ESNs (*r* = 0.28, *p* = 0.02) (**Figure 6C**) (Spearman rank tests), although responses in USNs were of lower magnitude than ESNs and seropositive individuals. There were no T-cell positive USNs under the age of 20, but several T-cell positive ESNs under the age of 20, suggesting that exposure to SARS-CoV-2 may generate T-cell immunity in children and adolescents who otherwise do not display cross-reactive cellular immunity.

**Figure 7:**
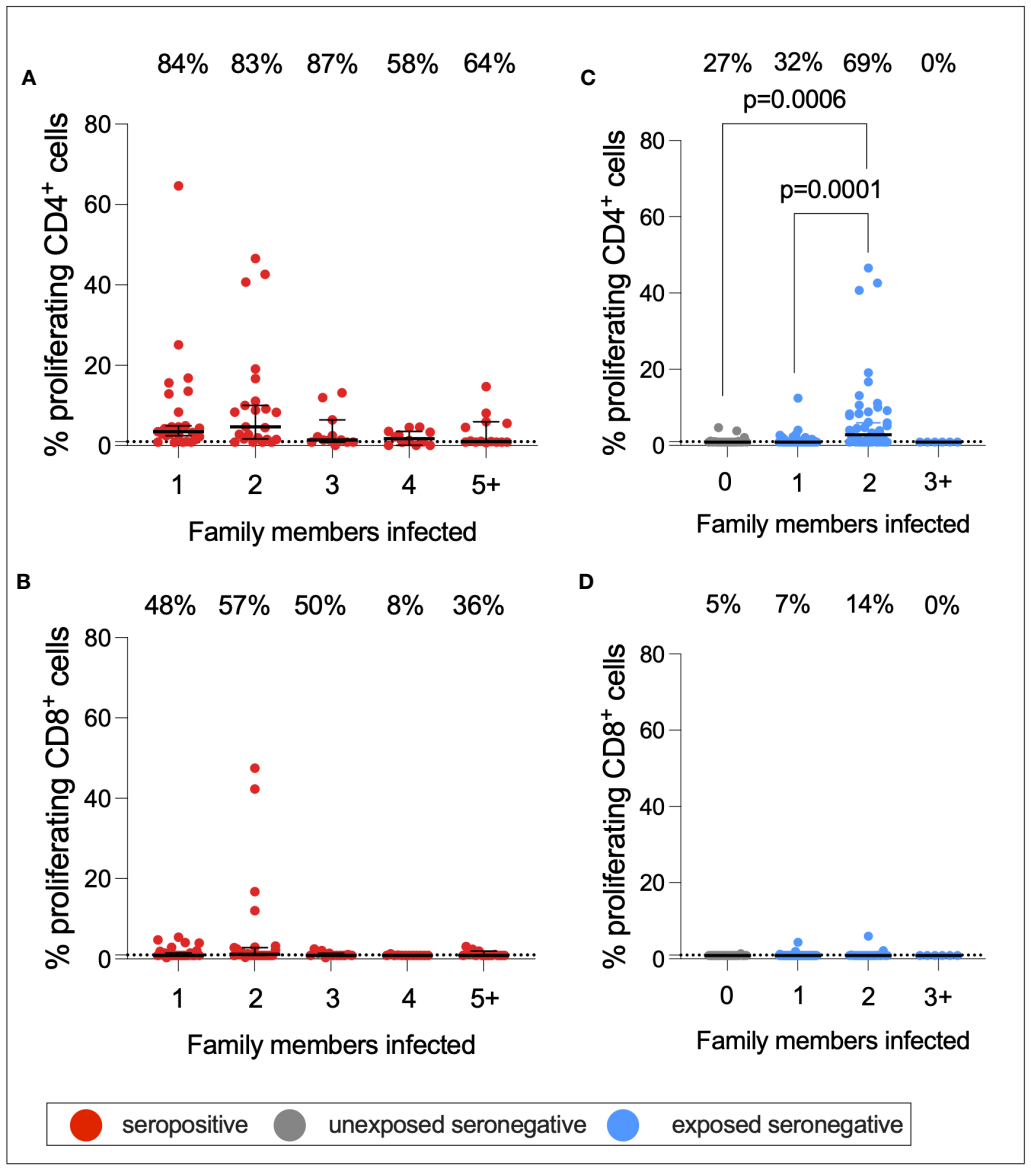
S1-specific T-cell responses by number of family members infected. CD4+ **(A)** and CD8+ **(B)** T-cell responses in seropositive (red) individuals with 1–5+ family members infected. CD4+ **(C)** and CD8+ **(D)** T-cell responses in USN (gray) and ESN (blue) individuals with 0–5+ family members infected. *p*-values refer to Mann–Whitney test values. Values below 1% were given nominal values of 0.9%. Dotted lines refer to cutoffs as determined previously (11). Percentages refer to the number of individuals with responses above 1% proliferation.

As described above, a greater proportion of seropositive individuals in a household was associated with an increased risk of infection. This also raised the question of whether a greater number of seropositive family members would be associated with stronger T-cell immunity in ESNs. To test this hypothesis, CD4+ and CD8+ T-cell responses targeting S1 were compared between seropositive individuals, USNs, and ESNs with different numbers of seropositive family members (**Figure 7**).CD4+ T-cell responses in seropositive individuals with different numbers of family members were significantly different (*p* = 0.01, Kruskal–Wallis test) with a trend of increased T-cell immunity in seropositive individuals with fewer infected family members, although no pairwise comparison was significant by Dunn’s multiple comparison test (**Figures 7A, B**). However, S1-specific CD4+ responses in ESNs with two infected family members were significantly higher than in USNs (*p* = 0.0001, Kruskal–Wallis test with Dunn’s multiple comparisons) (**Figure 7C**). There was no significant difference in T-cell response between individuals with no (*n* = 22) and one (*n* = 43) infected family member (*p* = 0.5, Mann–Whitney test). Of note, ESNs with two infected family members (*n* = 45) made significantly stronger T-cell responses to S1 than ESNs with one infected family member (*p* = 0.004, Kruskal–Wallis test with Dunn’s multiple comparisons) (**Figure 7C**), providing a direct link between the intensity of viral exposure and the strength of cellular immunity in seronegative individuals. Responses appeared lower in ESNs with 3+ members infected, although this is likely an artifact of the small numbers of individuals in this group (*n* = 6).

To further evaluate the nature of the T-cell response in ESNs, and to assess whether T-cell responses in ESNs target more structural antigens than USNs, the ratio between T cells targeting SARS-CoV-2 structural proteins (SPs) to NSPs was calculated for seropositive individuals, ESNs, and USNs, for both CD4+ and CD8+ T cells (**Supplementary Figure 5A**) Seropositive individuals had significantly higher SP : NSP ratios than ESNs for both CD4+ (1.8 vs. 0.6, *p* < 0.0001) and CD8+ (0.97 vs. 0.33, *p* < 0.0001) T cells (Mann–Whitney tests). There was no significant different in SP : NSP ratio between ESNs and USNs. To assess the levels of circulating HCoV-specific T cells in these individuals, proliferation assays were carried out on a subset of individuals (*n* = 35) using pools of peptides spanning the S2 region of S from HCoV-OC43 and HCoV-HKU1 (**Supplementary Figures 5B, C**). Responses to these antigens were small, and there was no significant difference in T-cell responses to HCoV-OC43 or HCoV-HKU1 between seropositive individuals, ESNs or USNs, although there was a trend of greater CD4+ responses in seropositive individuals.

To test the hypothesis that elevated T-cell immunity in ESNs may be due to the expansion of pre-existing, cross-reactive T cells targeting conserved regions of SARS-CoV-2 S, T-cell responses to a pool of 63 peptides highly conserved between SARS-CoV-2 and endemic HCoVs, as defined by Mateus et al. (2020) (14), were compared between seropositive individuals, ESNs, and USNs (**Supplementary Figure 5D**) (14). Furthermore, to distinguish between the expansion of total S responses versus expansion of conserved responses specifically, the ratio of responses to conserved peptides to S1 response was calculated and compared between groups (**Supplementary Figure 5E**). There was no significant difference in the magnitude of responses to the conserved pool between seropositive individuals, ESNs, and USNs, although there was a trend towards stronger responses in seropositive individuals. Furthermore, there was no significant difference in conserved S:S1 ratio between seropositive individuals, ESNs, and USNs. Although these comparisons are between small numbers of individuals, this does not support a model of cross-reactive immunity to endemic HCoVs expanding upon SARS-CoV-2 exposure, instead suggesting that *de novo* responses may also play a part.

### Humoral immunity in exposed seronegative individuals

**Figure 8:**
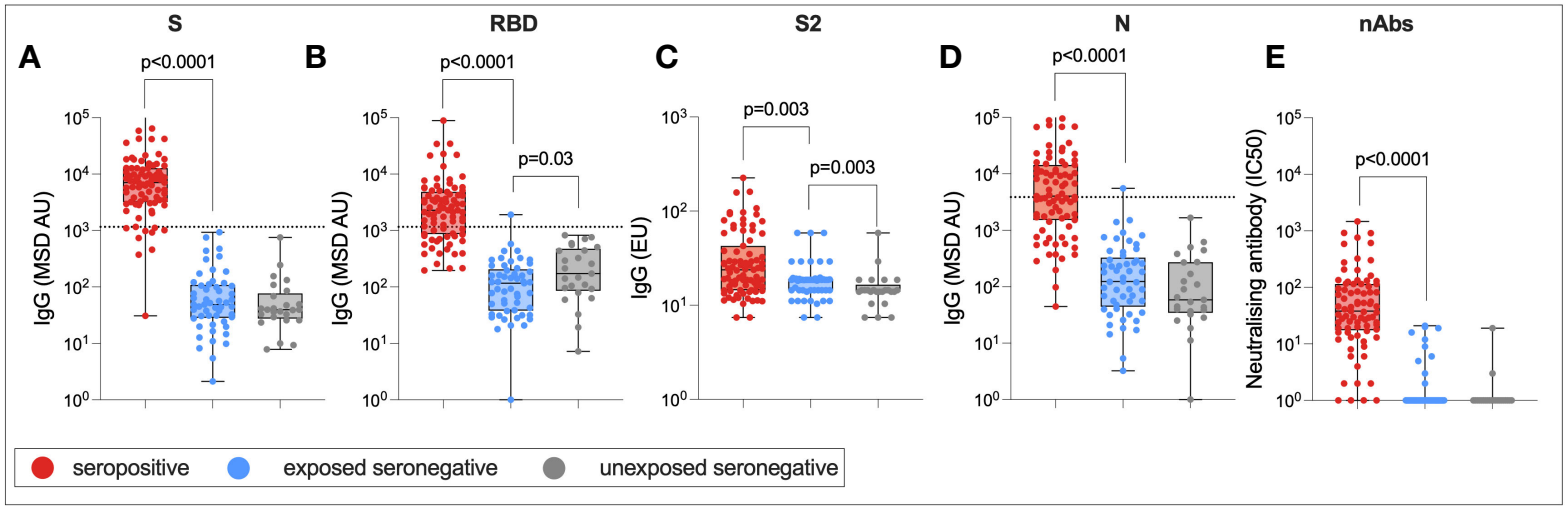
Humoral immune responses in ESNs. IgG targeting S **(A)**, RBD **(B)**, S2 **(C)**, and N **(D)**, and nAb titers **(E)** in seropositive (red), ESN (blue), and USN (gray) individuals. *p*-values refer to Mann–Whitney test values. Dotted lines represent seropositivity cutoffs as previously determined (12).

Finally, to assess whether there was any difference in humoral immune responses between USNs and ESNs, total IgG targeting SARS-CoV-2 S RBD, S2, and N, as well as nAb titers, were compared between seropositive, ESN, and USN individuals (**Figure 8**). By definition, responses to S, RBD, S2, and N were significantly higher in seropositive individuals compared to ESN individuals (S: 7,092 vs. 49, *p* < 0.0001; RBD: 2,288 vs. 116, *p* < 0.0001; S2: 24 vs. 19, *p* = 0.003; N: 4,043 vs. 124, *p* < 0.001; Mann–Whitney tests). nAb responses were also significantly higher in seropositive individuals compared to ESNs (38 vs. 1, *p* < 0.0001, Mann–Whitney test). While responses to S2 were higher in ESNs (18 vs. 14, *p* = 0.003, Mann–Whitney test) and IgG responses to total S were not significantly different between ESN and USN individuals, unexpectedly, responses to RBD were higher in USNs (173 vs. 116, *p* = 0.03, Mann–Whitney test). However, taking correction for multiple tests into account, this difference in antibody responses to RBD loses statistical significance. This may also reflect experimental noise as the difference is below the threshold for a positive response. An alternative explanation is that the expansion of cross-reactive S2-specific antibodies in ESNs leads to a reduction in antibody response to less conserved regions such as the RBD through competition for resources between B cells. However, it is perhaps more likely that this finding is artifactual.

## Discussion

Here, we describe transmission dynamics and immune responses in family groups sampled during the early stage of the pandemic, where SARS-CoV-2 infections were occurring in immunologically naïve hosts through ongoing household transmission. Notably, we describe elevated T-cell immunity in exposed seronegative individuals that is positively associated with the number of seropositive individuals in a household.

Over half of the cohort was seropositive, suggesting that transmission between family members was consistently occurring. Correlates of protection for SARS-CoV-2 are still uncertain, and it is unclear whether total IgG or nAb response is a better marker for protective immunity. However, the significantly elevated nAb response in seropositive individuals demonstrates that total IgG and nAb responses are well associated following natural infection, as shown previously (15), even many months into convalescence. nAb titers following infection have been shown to be durable and detectable after 8 months as supported here, a mean of 7 months post-infection (16). CD4+ T-cell responses were identified in most seropositive individuals as well as some seronegative individuals in this cohort. In seropositive individuals, responses primarily targeted S1 and S2 regions of S, with some individuals generating responses to M and N as well as NSP3B. Seronegative individuals also targeted these NSPs, although the majority of the response was specific for S1 and S2. Confirming previous findings by Ogbe et al. (12), few seronegative individuals generated T-cell responses to M and N (11). Antigenic targets of T-cell responses in unexposed individuals were well characterized in a study by Mateus et al. (14), which also identified S as the primary target of T-cell responses in pre-pandemic samples (54% of the total positive T-cell response). Eleven percent of this response targeted the RBD region of S, while 44% targeted non-RBD (14).

The findings from multiple linear regression that T-cell immunity was associated with older age and male sex are of note. Takahashi et al. (2020) identified higher T-cell activation in female than in male patients, although poor T-cell immunity was associated with worse COVID-19 outcomes in male patients only, and T-cell response was negatively correlated with age in male patients only (17). The discrepancy between these findings and those published previously may lie in the different cellular assays used, or the fact that these samples were taken months into convalescence. The cohorts in Takahashi et al. (2020) were sampled following a positive PCR test approximately 1 week after symptom onset, were more diverse in terms of ethnicity, and were all adults. Furthermore, the authors employed T-cell surface and intracellular staining rather than proliferation assays to investigate cellular immunity (17). Male patients may generate more proliferative memory responses to SARS-CoV-2 while female patients might express higher levels of activation markers.

It was also identified that S1-specific CD4+ T-cell immunity was positively correlated with age in both USNs and ESNs. This is of note, particularly as cellular immune responses to SARS-CoV-2 in infected children have been demonstrated to be of similar magnitude and functionality compared to adults (18). In this study, the correlation was lost following SARS-CoV-2 infection. It could therefore be inferred that this trend reflects the accumulation of cross-reactive T cells targeting endemic betacoronaviruses over an individual’s lifetime, which are less frequently observed in children. However, following SARS-CoV-2 infection, cross-reactive immunity plays a smaller role and therefore the correlation between age and S1-specific T cells is lost. An alternative explanation is that this cross-reactivity is an artifact of using synthetic peptides.

Case studies of individual families provide an opportunity to assess immunity within families in more detail. In families all seropositive for SARS-CoV-2, humoral and cellular responses were varied. Previous studies have identified lower respiratory cycle threshold (Ct) values and higher plasma IgG in “high transmission” families where all individuals become infected compared to “low transmission” families where individuals were mixed serostatus (19). Nasopharyngeal sampling of this cohort during acute infection would have enabled this analysis and further provided an opportunity to assess what makes these “all seropositive” families all become infected, and whether this derives from virus-associated factors such as viral load.

In families all seronegative for SARS-CoV-2, cellular immunity was observed, but only in a few individual parents. In contrast, in mixed serostatus families where parents were either both seropositive or serodiscordant, cellular immunity was also present in seronegative children. This raised the question of whether intrafamilial exposure to SARS-CoV-2 could expand cellular immunity to SARS-CoV-2. This has been demonstrated previously in the context of HCWs (9, 11, 20) and in a small cohort of serodiscordant couples (10). However, this cohort is one of the largest to assess immune responses in ESN family members. In this cohort, elevated CD4+ T-cell responses in ESNs targeted S1, with a trend towards greater responses in S2, M, and N. This is in accordance with previous findings that demonstrate enhanced S-, M-, and N-specific immunity in ESN HCWs (11). However, elevated T-cell immunity to non-S regions of the SARS-CoV-2 proteome has also been identified in ESNs (20). In the data mentioned herein, there was a trend towards stronger T-cell immunity in ESNs for some NSPs such as ORF3, ORF8, NSP3A, and NSP3C, compared to USNs. However, T-cell responses targeting the RTC (NSP7, NSP12, and NSP13) were not significantly elevated in ESNs compared to USNs, in contrast to published findings (9).

Although both CD4+ and CD8+ responses were significantly more structurally targeted in seropositive individuals, there was no difference in the SP : NSP ratio between ESNs and USNs in this cohort. This suggested that ESNs generate a T-cell response more reminiscent of USNs than of seropositive individuals. A skew towards T cells targeting NSPs during ZIKV infection in individuals previously exposed to DENV has been reported; this is due to high homology between flavivirus NSPs (21). Here, though responses to structural proteins such as S1 are significantly higher in ESNs than in USNs, the overall SP : NSP ratio does not differ significantly. There was also no evidence of increased HCoV-specific immunity, or immunity to conserved regions of S, in ESNs compared to USNs. This casts doubt on whether cross-reactivity from endemic HCoVs is the sole source of T-cell responses in ESNs. Another explanation could be a combination of pre-existing cellular immunity combined with low-level *de novo* responses to novel SARS-CoV-2 epitopes upon low-dose viral exposure. It is unclear to what extent this immunity is protective; cross-reactive T cells have been associated with protection against infection with SARS-CoV-2 (22), but further investigation through SARS-CoV-2 challenge is required to confirm these findings.

**Figure 9:**
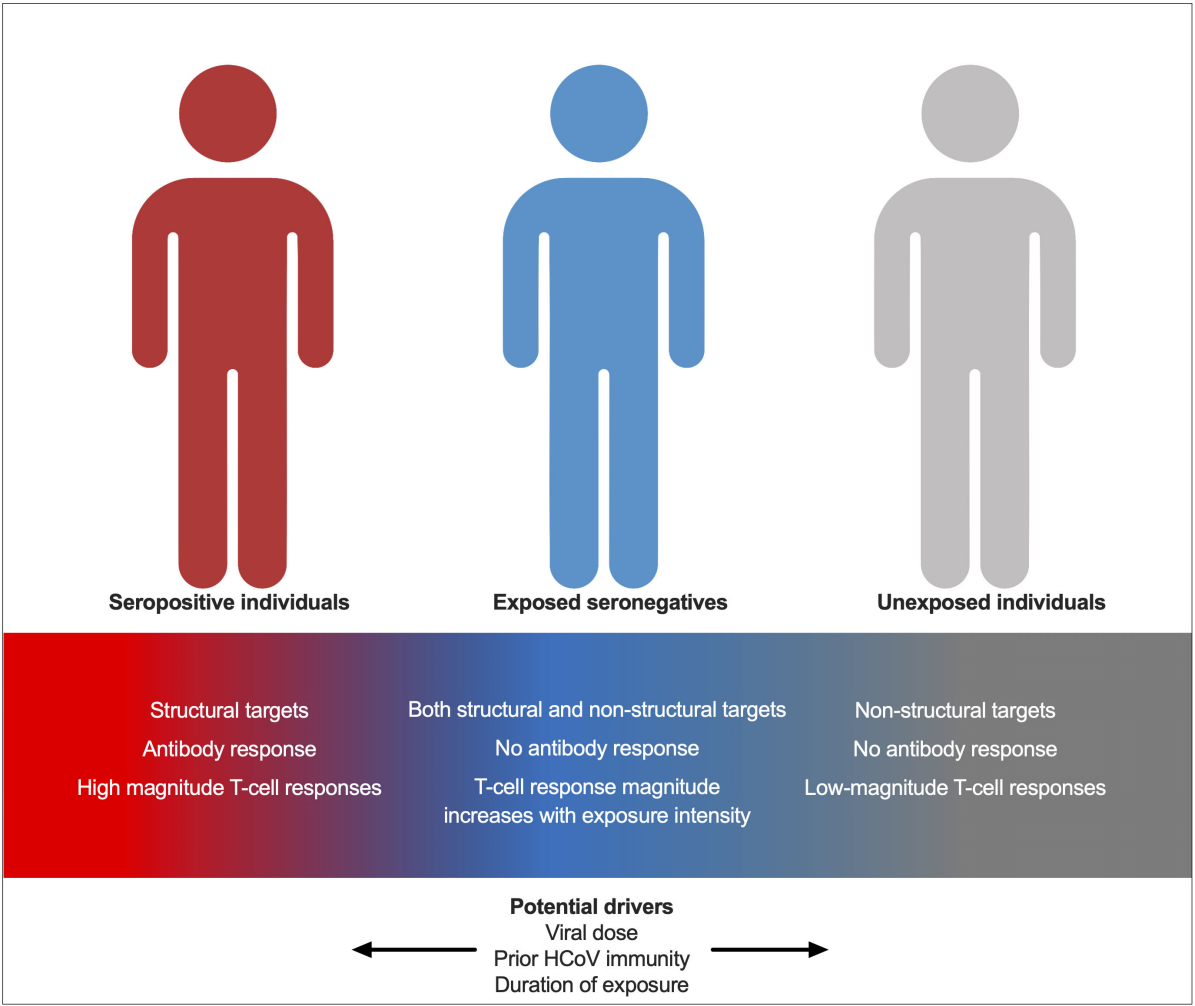
A model for immunity in seropositive individuals, ESNs, and USNs. Individuals represent points along a spectrum from USN to seropositive, modulated by viral dose, prior immunity to endemic HCoVs, and duration of exposure.

The role of exposure intensity in ESNs has not been studied for SARS-CoV-2. Here, we describe increased T-cell responses in seronegative individuals with two seropositive family members compared to those with only one seropositive family member, indicating that enhanced exposure intensity is associated with stronger cellular immunity. This may be due to an increased viral dose. The role of dose in ESNs has been studied for HCV and simian immunodeficiency virus (SIV) in non-human primates: transient T-cell responses were demonstrated when two chimpanzees were exposed to increasing doses of HCV at 6-month intervals. Twelve months later, when both chimpanzees were exposed to a 10-fold greater dose of virus, the chimpanzee with consistently stronger T-cell responses cleared infection while the other developed chronic disease (23). Furthermore, macaques exposed to infectious doses of SIV seroconverted but generated weak cellular responses, while those exposed to sub-infectious doses generated cellular responses only (24). These findings suggest that dose may factor into which arm of adaptive immunity dominates upon viral exposure. Similar challenge studies in primates or humans exposed to different doses of SARS-CoV-2 would be necessary to make conclusions about the role of dose in SARS-CoV-2 ESNs. However, the findings described herein suggest that increased dose may promote enhanced cellular immunity in ESNs, while perhaps pushing individuals towards a dose threshold, the surpassing of which leads to infection and seroconversion. An alternative explanation is that an increased number of seropositive individuals within a family increases the duration, rather than the dose, of viral exposure in ESNs. An increased duration of HCV exposure is associated with stronger T-cell responses in ESN injection drug users and is also associated with an increased durability of response (25). ESNs likely represent a spectrum between USN and seropositive individuals, with their position upon the spectrum determined by prior exposure to HCoVs, viral dose, and exposure intensity (**Figure 9**).

The study has several limitations. The collection of samples in convalescence adds the potential confounding element of response waning over time. An attempt to correct for this using multiple linear regression was carried out, but sampling during acute disease or at a fixed time after infection would have enabled more robust comparisons between individuals. Similarly, sampling during acute disease and nasopharyngeal sampling would have allowed for PCR confirmation of infection rather than using serological data as a marker of prior infection. This is a significant limitation of the study, as ESNs could potentially reflect once-seropositive individuals whose antibodies have waned below the cutoff for seropositivity. However, using nAb response as a determinant of seropositivity instead did not change the results of the analysis, and there was no correlation between T-cell response and humoral response in ESNs, suggesting that ESN individuals with large S1-specific CD4+ responses are not simply convalescent individuals whose humoral immunity has waned below the threshold for seropositivity. Furthermore, although some individuals classed as seropositive by ELISA were seronegative by MSD, classifying seropositivity using the results of the MSD assay or the pseudovirus neutralization assay rather than the ELISA assay did not significantly impact the finding that ESNs generate greater responses to S1 and S2 than USNs, supporting the accuracy of ELISA data to determine seropositivity (**Supplementary Figure 6**). Finally, sampling of individuals at later time points would also have facilitated an assessment of the potential protective capacity of cellular immunity, as well as its impact on vaccine response.

Overall, this study demonstrates intrafamilial transmission of SARS-CoV-2 as a major route of infection early in the pandemic when individuals were predominantly SARS-CoV-2-naïve. We show an increased risk of infection, and an increased T-cell response in seronegative family members, when more family members become infected. Sex- and age-related differences in immune response appear minimal, although regression analysis identifies an association between older age, male sex, and increased T-cell immunity. Finally, T-cell immunity in ESNs does not appear to originate solely from cross-reactive responses to endemic HCoVs but may also be generated *de novo* upon exposure to SARS-CoV-2. These findings have implications for defining SARS-CoV-2 correlates of protection in the future, as T-cell immunity may be protective when vaccine-induced humoral immunity has waned.

## Data availability statement

The raw data supporting the conclusions of this article will be made available by the authors, without undue reservation.

## Ethics statement

The studies involving humans were approved by University of Oxford Central University Research Ethics Committee. The studies were conducted in accordance with the local legislation and institutional requirements. Written informed consent for participation in this study was provided by the participants’ legal guardians/next of kin.

## Author contributions

CJ: Conceptualization, Formal Analysis, Investigation, Writing—Original Draft Preparation, Review & Editing, and Visualization. EA: Investigation, Data Curation, Writing—Review & Editing, and Project Administration. AC: Investigation, Data Curation, Writing—Review & Editing, and Project Administration. CD: Investigation, Data Curation, and Writing—Review & Editing. ME: Investigation, Data Curation, and Writing—Review & Editing. C-PH: Investigation, Data Curation, and Writing—Review & Editing. AJ: Resources and Writing—Review & Editing. NL: Investigation and Writing—Review & Editing. SL: Formal Analysis, Investigation, and Writing—Review & Editing. AO: Investigation and Writing—Review & Editing. OS: Investigation and Writing—Review & Editing. DS: Conceptualization and Writing—Review & Editing. OBS: Resources and Writing—Review & Editing. LS: Resources, Methodology, and Writing—Review & Editing. CT: Resources, Writing—Review & Editing, and Methodology. LT: Supervision and Writing—Review & Editing. EB: Conceptualization and Writing—Review & Editing. SD: Supervision and Writing—Review & Editing. MC: Supervision, Writing—Review & Editing, and Methodology. PK: Supervision, Writing—Review & Editing, Conceptualization, and Methodology. CC: Conceptualization, Methodology, and Writing—Review & Editing. PG: Conceptualization, Methodology, Resources, Writing—Review & Editing, Supervision, Project Administration, and Funding Acquisition. LJ: Conceptualization, Methodology, Resources, Project Administration, Funding Acquisition, and Writing—Review & Editing. All authors contributed to the article and approved the submitted version.

## Supplementary material

Supplementary Figure 1 Family types. Family ID numbers are shown below each pedigree chart. Example families where all individuals became infected **(A)**. Families where all remained seronegative **(B)**. Families where only the father or an adult male were infected **(C)**. Families where only the mother was infected **(D)**. Families where only children were infected **(E)**. Families where one parent and a child/children were infected **(F)**. Families where both parents and a child/children were infected **(G)**. Squares are male patients; circles are female patients. Seropositive individuals are shown in red; seronegative individuals are in gray.

Supplementary Figure 2 Family types C, D, and E. Anti-S IgG, nAb responses, CD4+ T-cell responses and CD8+ responses in a household group consisting of a seropositive male, a seronegative male, and two seronegative female patients **(A)**. IgG, nAb, and T-cell responses in a household group consisting of a seropositive mother and seronegative father, daughter, and son **(B)**. IgG, nAb, and T-cell responses in a household group consisting of seronegative parents, two seropositive daughters, and two seronegative daughters **(C)**. Seropositive family members are outlined in red. Male patients are squares; female patients are circles. Asterisks refer to symptomatic individuals. Proliferation values below 1% were given nominal values of 0.9%.

Supplementary Figure 3 Family types F and G. Anti-S IgG, nAb responses, CD4+ T-cell responses, and CD8+ responses in a family group consisting of a seropositive father, a seronegative mother, two seronegative sons, one seronegative daughter, and a seropositive son **(A)**. IgG, nAb, and T-cell responses in a family group consisting of seropositive parents, two seropositive sons, two seropositive daughters, and a seronegative daughter **(B)**. Male patients are squares; female patients are circles. Seropositive family members are outlined in red. Asterisks refer to symptomatic individuals. Proliferation values below 1% were given nominal values of 0.9%.

Supplementary Figure 4 Correlations between S1-specific CD4+ response and other immune parameters. Correlation between S1-specific CD4+ T cells and S2-specific CD4+ T cells **(A)**, nAbs **(B)**, and anti-S (IgG) **(C)** in ESNs. *R*- and *p*-values refer to Spearman rank correlation values. Proliferation values below 1% were given nominal values of 0.9%. Dotted lines refer to the cutoff for T-cell positivity of 1%.

Supplementary Figure 5 T-cell responses in ESNs are not associated with enhanced HCoV-specific immunity. Ratio of CD4+ and CD8+ SP to NSP responses **(A)**, T cells targeting HCoV-OC43 S2 **(B)**, T cells targeting HCoV-HKU1 S2 **(C)**, T cells targeting a pool of 63 conserved peptides **(D)**, and ratio of response to the conserved pool against total S1 response **(E)** in seropositive (red), ESN (blue), and USN (white) individuals. *P*-values refer to Mann–Whitney test values. Proliferation values below 1% were given nominal values of 0.9%.

Supplementary Figure 6 T-cell responses in seropositive, ESN, and USN individuals as defined by MSD or nAb serostatus. % proliferating CD4+ T cells targeting S1 and S2 in seropositive (red), ESN (blue), and USN (gray) individuals, as defined by MSD serostatus **(A)** with an AU > 1,160, or nAb serostatus **(B)** with an IC_50_ > 0. *p*-values refer to Mann–Whitney test values. Proliferation values below 1% were given nominal values of 0.9%.

Supplementary Figure 7 Gating strategy for proliferation assay.
