# Supplementary figures and images for: Cellular immunity to SARS-CoV-2 following intrafamilial exposure in seronegative family members
Source: Front Immunol. 2023 Aug 29;14:1248658. doi: 10.3389/fimmu.2023.1248658 (PMC10497976; doi:10.3389/fimmu.2023.1248658)

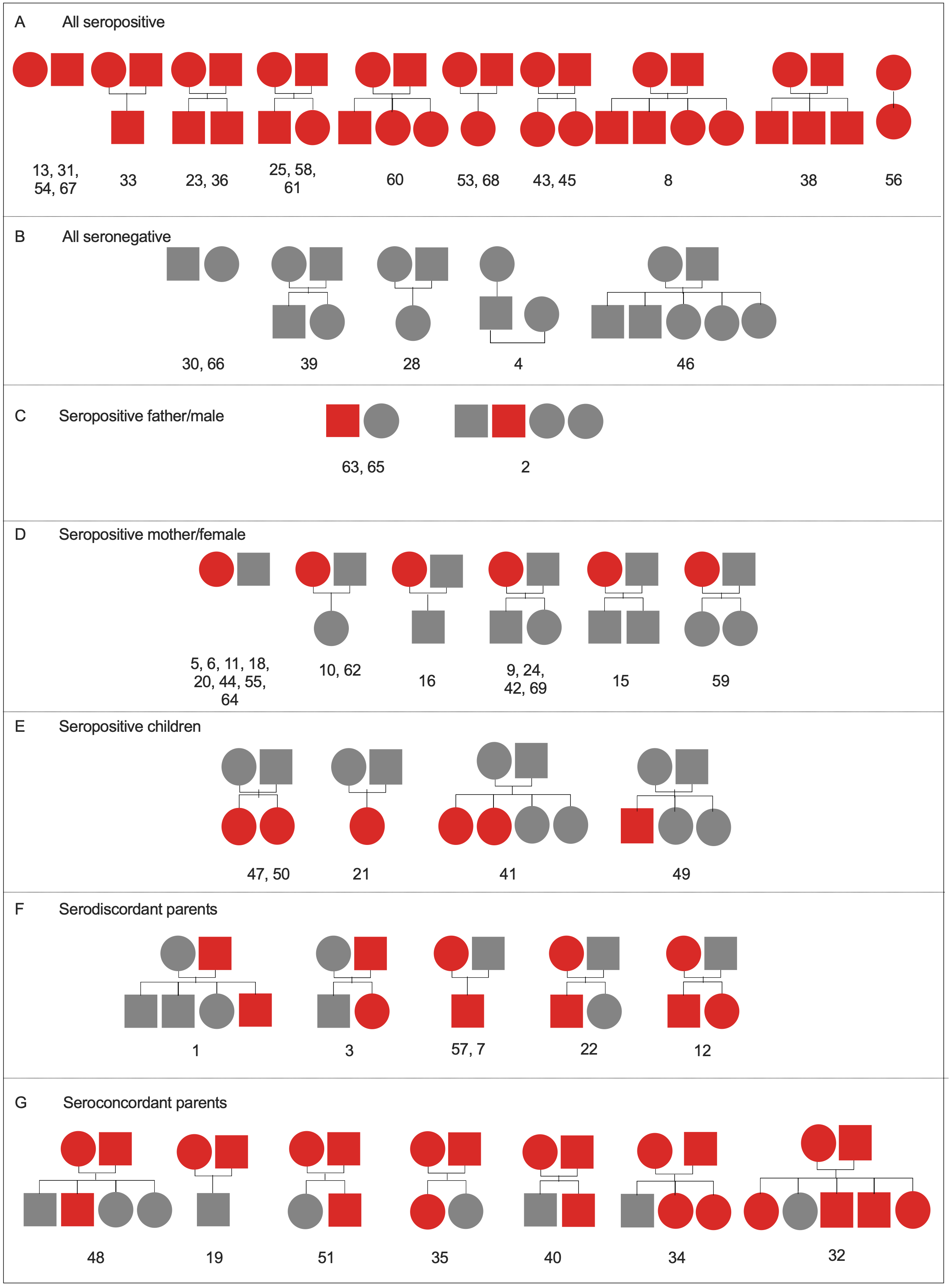

Supplement: Supplementary Figure 1 — Family types. Family ID numbers are shown below each pedigree chart. Example families where all individuals became infected (A). Families where all remained seronegative (B). Families where only the father or an adult male were infected (C). Families where only the mother was infected (D). Families where only children were infected (E). Families where one parent and a child/children were infected (F). Families where both parents and a child/children were infected (G). Squares are male patients; circles are female patients. Seropositive individuals are shown in red; seronegative individuals are in gray. [file Image_1.jpeg]

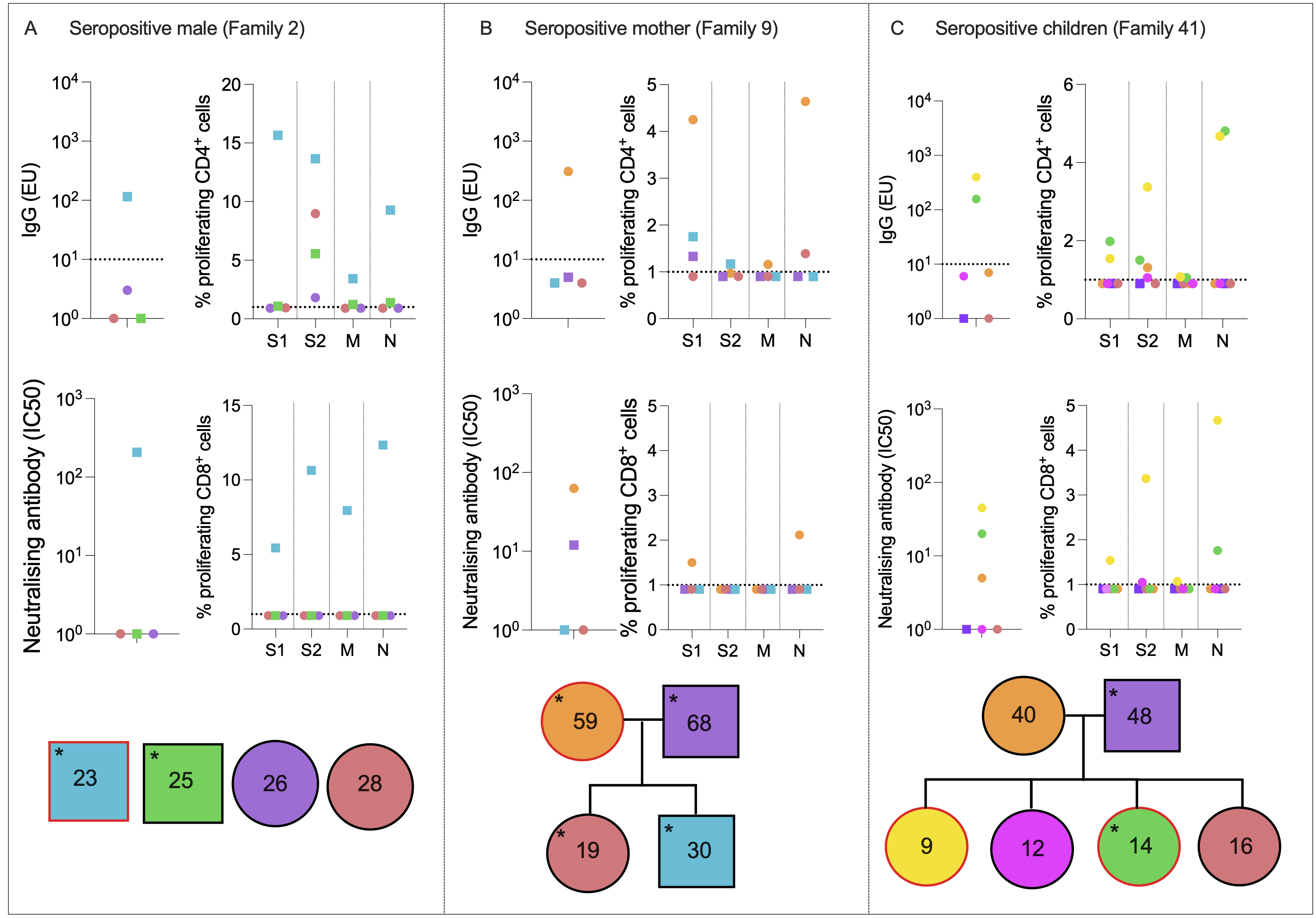

Supplement: Supplementary Figure 2 — Family types C, D, and E. Anti-S IgG, nAb responses, CD4+ T-cell responses and CD8+ responses in a household group consisting of a seropositive male, a seronegative male, and two seronegative female patients (A). IgG, nAb, and T-cell responses in a household group consisting of a seropositive mother and seronegative father, daughter, and son (B). IgG, nAb, and T-cell responses in a household group consisting of seronegative parents, two seropositive daughters, and two seronegative daughters (C). Seropositive family members are outlined in red. Male patients are squares; female patients are circles. Asterisks refer to symptomatic individuals. Proliferation values below 1% were given nominal values of 0.9%. [file Image_2.jpeg]

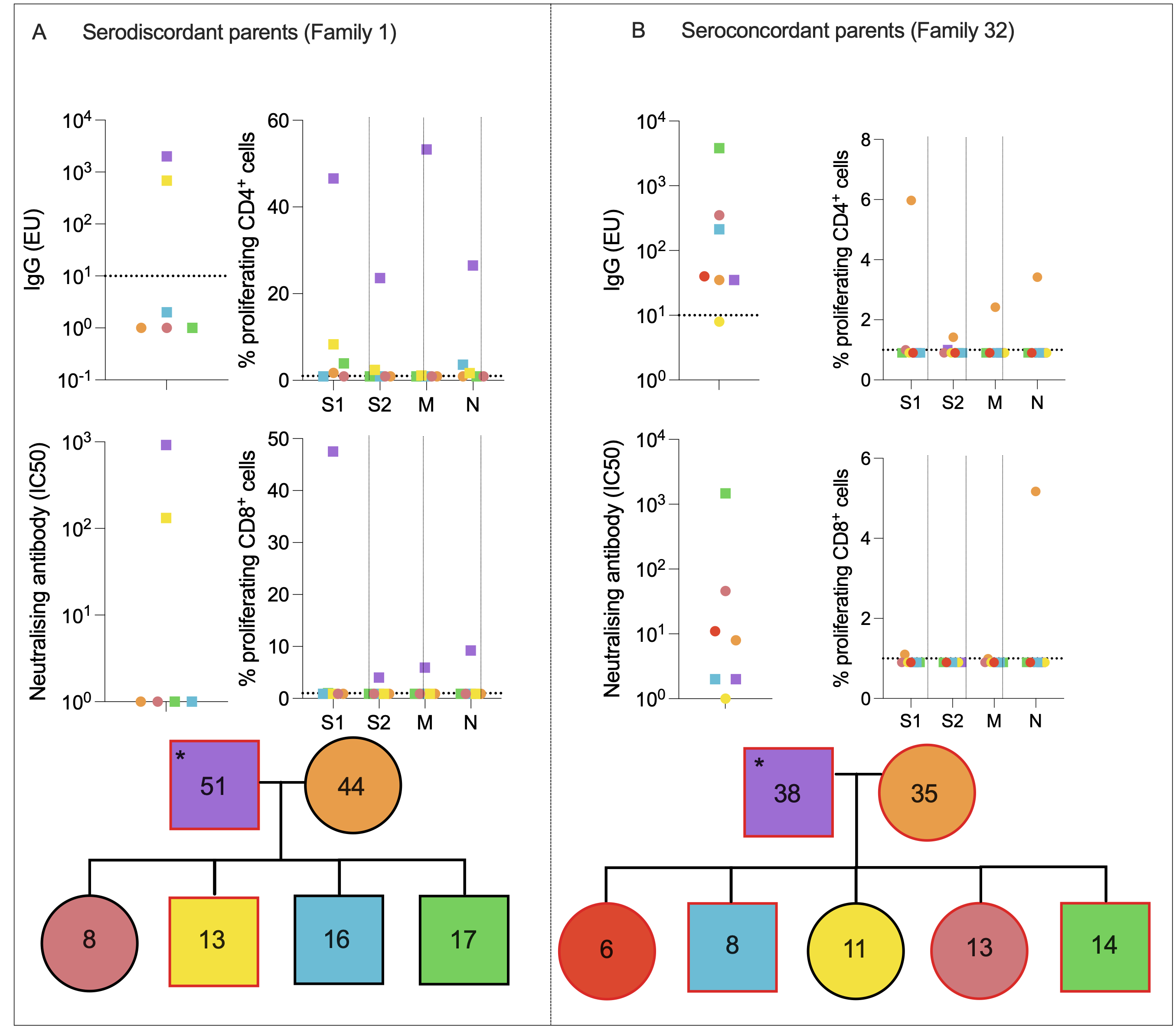

Supplement: Supplementary Figure 3 — Family types F and G. Anti-S IgG, nAb responses, CD4+ T-cell responses, and CD8+ responses in a family group consisting of a seropositive father, a seronegative mother, two seronegative sons, one seronegative daughter, and a seropositive son (A). IgG, nAb, and T-cell responses in a family group consisting of seropositive parents, two seropositive sons, two seropositive daughters, and a seronegative daughter (B). Male patients are squares; female patients are circles. Seropositive family members are outlined in red. Asterisks refer to symptomatic individuals. Proliferation values below 1% were given nominal values of 0.9%. [file Image_3.jpeg]

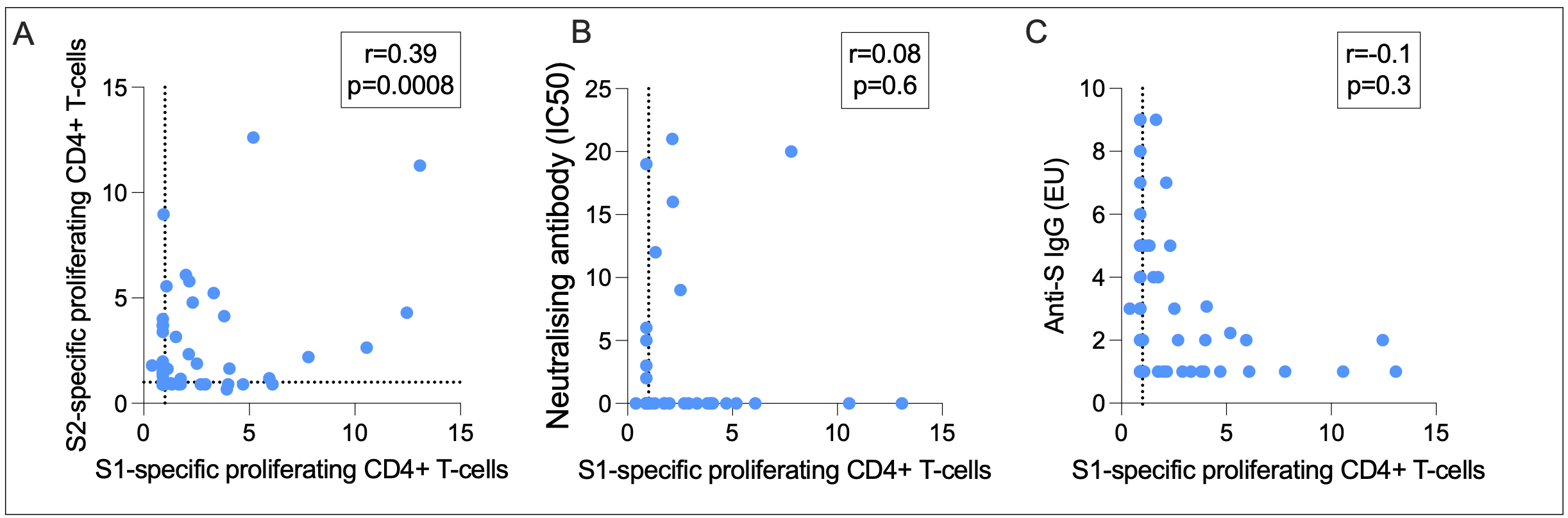

Supplement: Supplementary Figure 4 — Correlations between S1-specific CD4+ response and other immune parameters. Correlation between S1-specific CD4+ T cells and S2-specific CD4+ T cells (A), nAbs (B), and anti-S (IgG) (C) in ESNs. R- and p-values refer to Spearman rank correlation values. Proliferation values below 1% were given nominal values of 0.9%. Dotted lines refer to the cutoff for T-cell positivity of 1%. [file Image_4.jpeg]

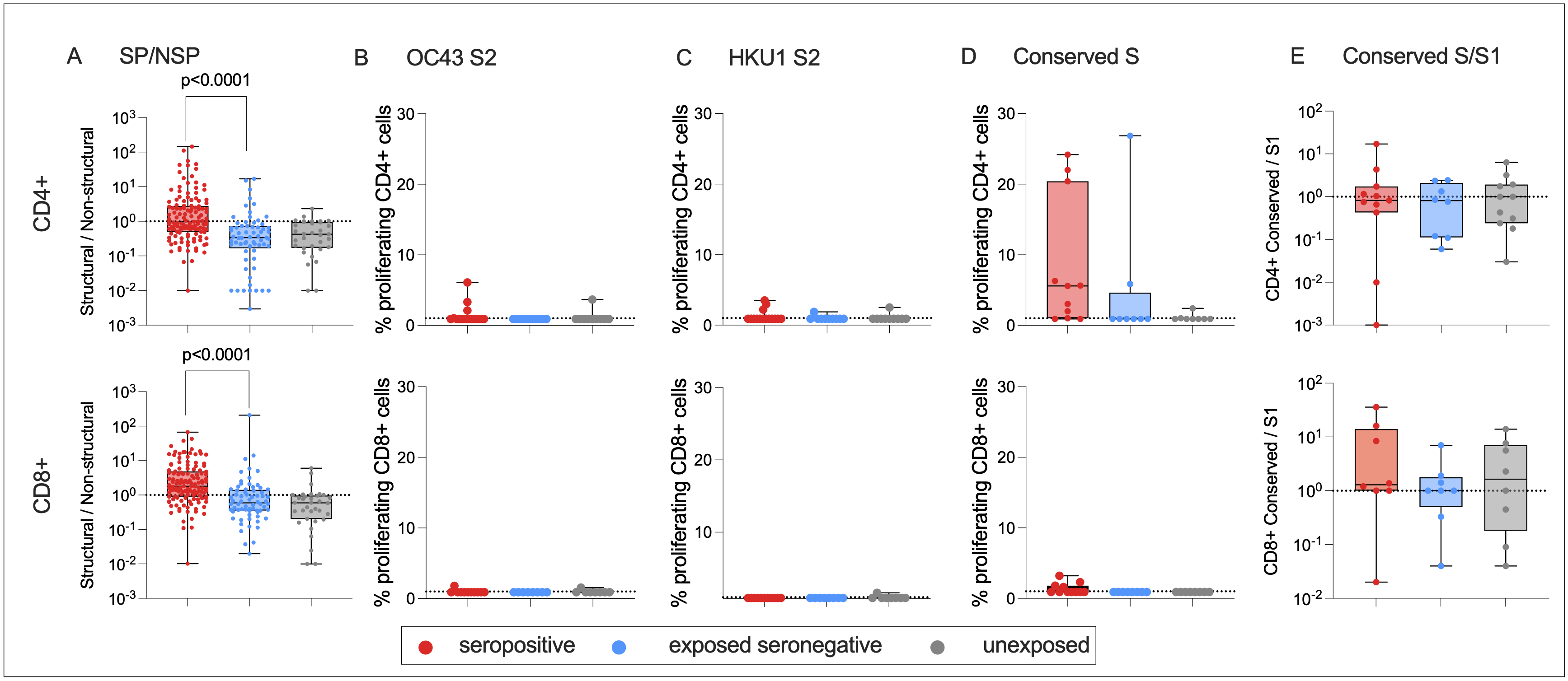

Supplement: Supplementary Figure 5 — T-cell responses in ESNs are not associated with enhanced HCoV-specific immunity. Ratio of CD4+ and CD8+ SP to NSP responses (A), T cells targeting HCoV-OC43 S2 (B), T cells targeting HCoV-HKU1 S2 (C), T cells targeting a pool of 63 conserved peptides (D), and ratio of response to the conserved pool against total S1 response (E) in seropositive (red), ESN (blue), and USN (white) individuals. P-values refer to Mann–Whitney test values. Proliferation values below 1% were given nominal values of 0.9%. [file Image_5.jpeg]

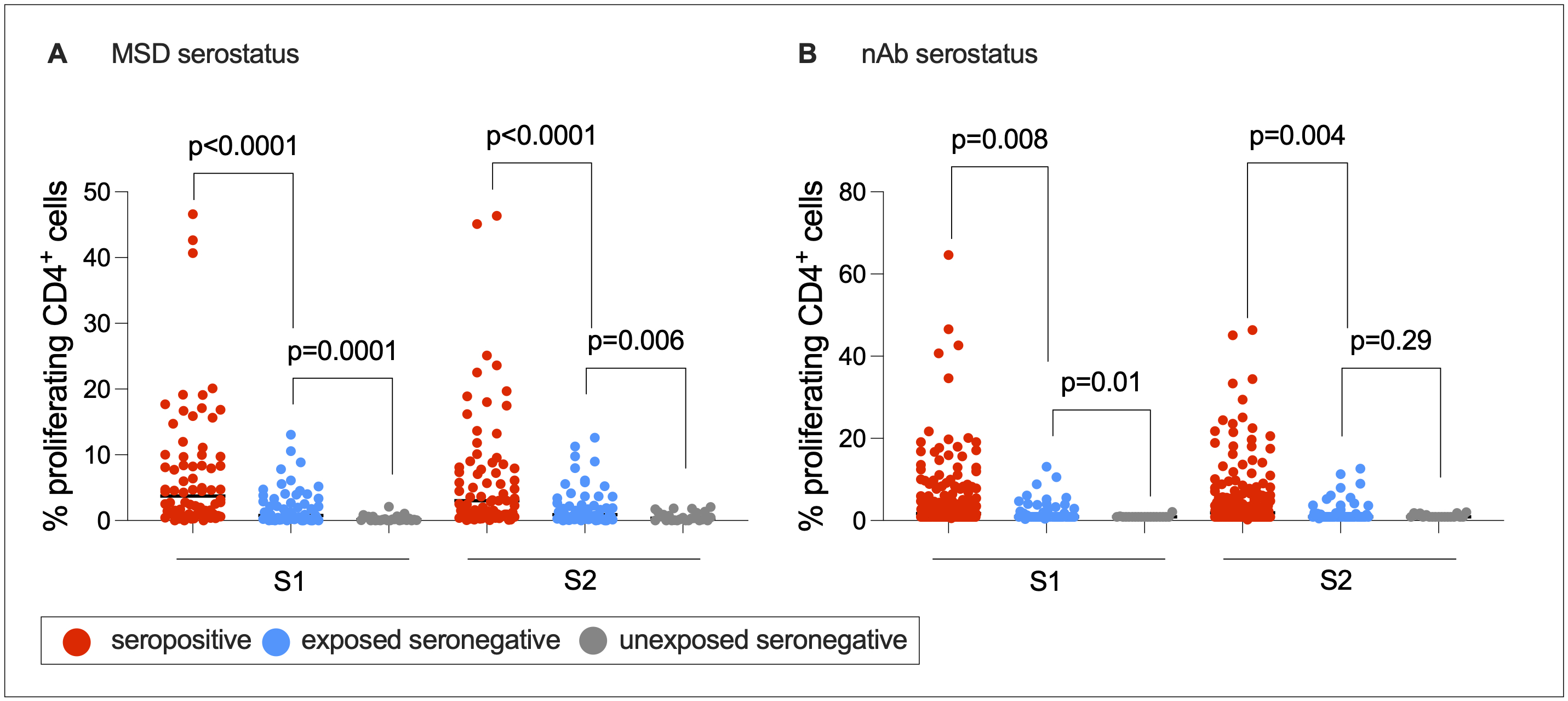

Supplement: Supplementary Figure 6 — T-cell responses in seropositive, ESN, and USN individuals as defined by MSD or nAb serostatus. % proliferating CD4+ T cells targeting S1 and S2 in seropositive (red), ESN (blue), and USN (gray) individuals, as defined by MSD serostatus (A) with an AU > 1,160, or nAb serostatus (B) with an IC50 > 0. p-values refer to Mann–Whitney test values. Proliferation values below 1% were given nominal values of 0.9%. [file Image_6.jpeg]

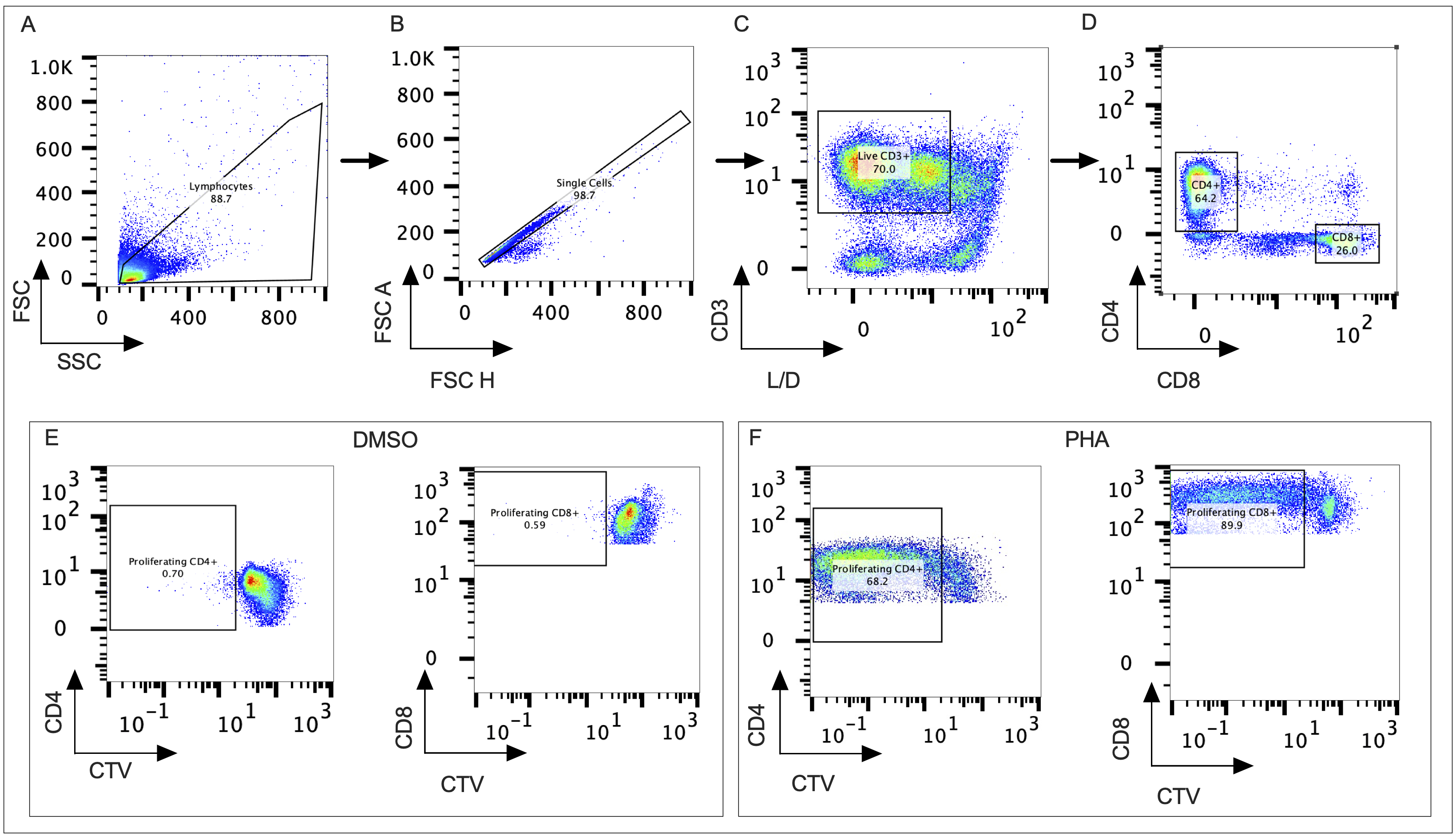

Supplement: Supplementary Figure 7 — Gating strategy for proliferation assay. [file Image_7.jpeg]
